# Genome-wide association studies revealed complex genetic architecture and breeding perspective of maize ear traits

**DOI:** 10.1186/s12870-022-03913-1

**Published:** 2022-11-18

**Authors:** Mita Khatun, Md Mamun Monir, Xiangyang Lou, Jun Zhu, Haiming Xu

**Affiliations:** 1 Institute of Crop Science and Institute of Bioinformatics, Zhejiang University, Hangzhou, 310058 China; 2 Department of Biostatistics, University of Florida, Gainesville, FL 32611 USA

**Keywords:** Maize ear traits, Mixed model, Dominance, Epistasis, Gene × environment interactions, Superior genotypes

## Abstract

**Background:**

Maize (*Zea Mays*) is one of the world’s most important crops. Hybrid maize lines resulted a major improvement in corn production in the previous and current centuries. Understanding the genetic mechanisms of the corn production associated traits greatly facilitate the development of superior hybrid varieties.

**Result:**

In this study, four ear traits associated with corn production of Nested Association Mapping (NAM) population were analyzed using a full genetic model, and further, optimal genotype combinations and total genetic effects of current best lines, superior lines, and superior hybrids were predicted for each of the traits at four different locations. The analysis identified 21–34 highly significant SNPs (−*log*_*10*_*P* > 5), with an estimated total heritability of 37.31–62.34%, while large contributions to variations was due to dominance, dominance-related epistasis, and environmental interaction effects (${h}_{D+}^2\hat{=}$ 14.06% ~ 49.28%), indicating these factors contributed significantly to phenotypic variations of the ear traits. Environment-specific genetic effects were also discovered to be crucial for maize ear traits. There were four SNPs found for three ear traits: two for ear length and weight, and two for ear row number and length. Using the Enumeration method and the stepwise tuning technique, optimum multi-locus genotype combinations for superior lines were identified based on the information obtained from GWAS.

**Conclusions:**

Predictions of genetic breeding values showed that different genotype combinations in different geographical regions may be better, and hybrid-line variety breeding with homozygote and heterozygote genotype combinations may have a greater potential to improve ear traits.

**Supplementary Information:**

The online version contains supplementary material available at 10.1186/s12870-022-03913-1.

## Background

The indigenous peoples of southern Mexico were the first to domesticate maize about 10,000 years ago, which has since become a staple food in many parts of the world [1, 2]. Maize is grown for corn oil, animal feed, corn starch, corn syrup, purslane corn, pod corn, popcorn, flour, and other corn-related products. Corn production has increased eight-fold in the last century as a result of advancement of hybrid varieties [3]. Genome-wide association studies (GWASs) of various experimental populations, such as the maize NAM population and large association panels, revealed the genetic architecture of several complex traits [4, 5], which provided important first insights into genetic mechanism. High chromosomal resolution of the NAM population promotes the identification of genes associated with complex traits [6], which could accelerate up marker-assisted breeding and help breeders to improve hybrid cron production.

Maize complex traits such as days to silk and leaf traits are controlled by polygenes that exhibit additive, dominance, epistasis, and gene-environment interaction effects [7, 8]. Many statistical methods, such as the additive model approach and the full genetic model approach, have been developed to uncover plant complex traits. However, most genetic association studies simply focus on additive effects, ignoring non-additive, epistasis, and gene-environment interaction effects, leading to a missing heritability problem that could have a large impact on phenotypic variability explanation [9]. Phenotypic variations of complex traits largely vary across multiple locations that might also be cause of missing heritability problem. Genetic effects of complex traits are different in multiple environments and the differences depend on geographical position, weather, soil, water etc. [10]. The full genetic model approach analyzes the factors to overcome the missing heritability problem. Genome-wide association study (GWAS) is a powerful tool that has been used to analyze complex traits of plants [11, 12]. By accounting for these underlying factors that may have significant impacts on complex traits of maize as in previous studies [13, 14], in this study, a GWAS is performed to dissect genetic architecture of four ear traits and further evaluate the potential of breeding improvements [14, 15]. By partitioning phenotypic variation into additive, dominance, epistasis, and gene × environment interactions, a mixed linear model approach implemented with *QTXNetwork* [16], was employed to dissect the genetic architecture of four maize ear traits (weight, length, rank number, and row number).

Maize ear traits were previously studied by various experimental crossings and multiple genes associated with them were discovered [17, 18]. No previous studies, however, examined dominance and dominance-related epistasis effects including (additive × dominance, dominance × additive, and dominance × dominance) on ear traits. Similar to the previous studies conducted for days to silk [14] and leaf traits [13] of maize NAM population, dominance and dominance-related epistasis effects were considered to evaluate impacts of these genetic factors on ear traits. Based on genetic effects estimated from the association study, the expected superior hybrid lines were designed and predicted in four different locations, Urbana, Aurora, Clayton, and Homestead of the United States.

## Results

### Phenotypic distributions of maize ear traits

**Fig. 1:**
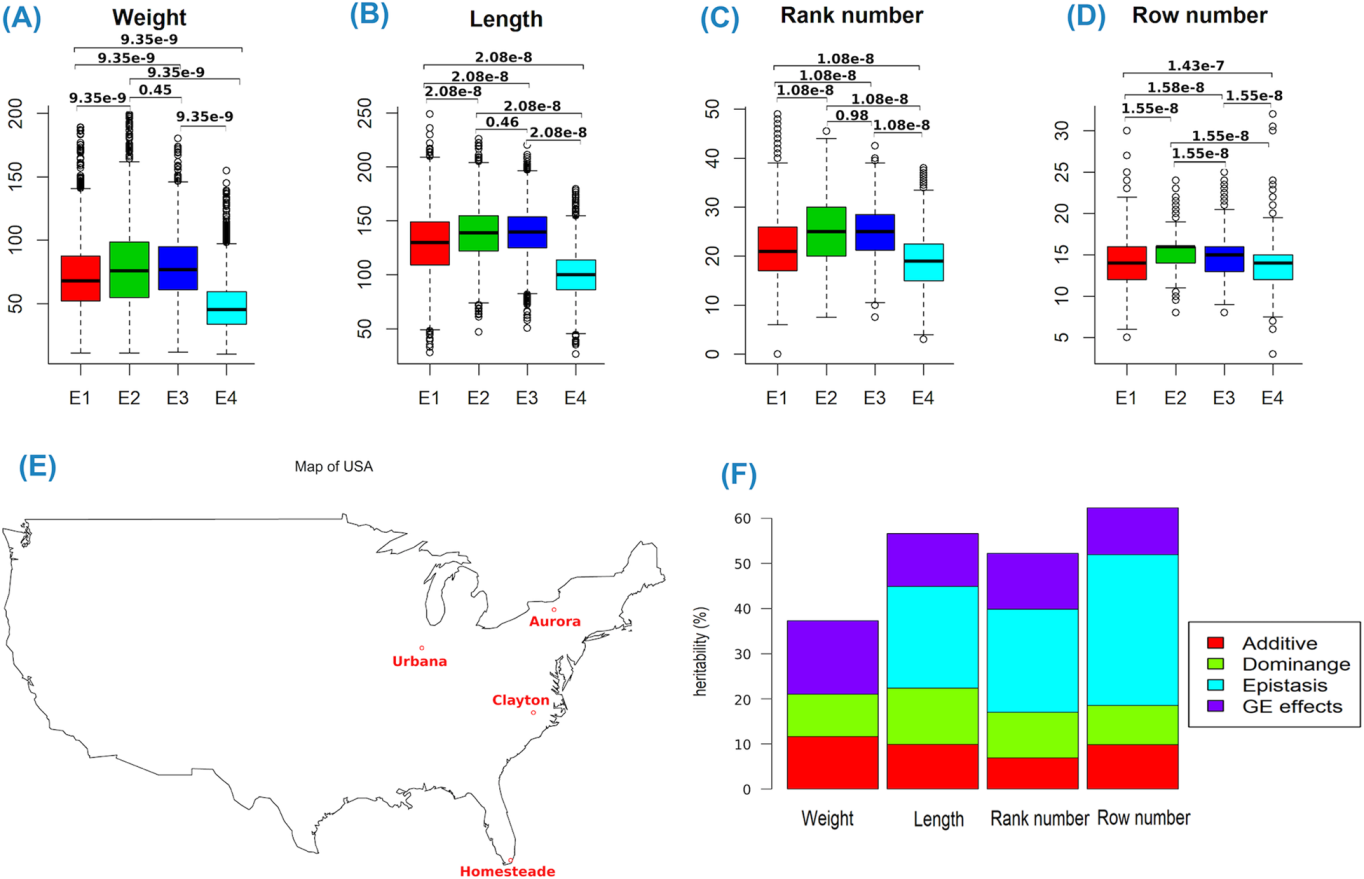
Phenotypic distributions, cultivating locations, and heritability estimation of maize ear traits. (**A**) Weight: ear weight, (**B**) Length: ear length, (**C**) Rank number: ear rank number, (**D**) Row number: ear row number, (**E**) Location map created using R package “maps”, and (**F**) Estimated heritability due to different genetic effect for each of the maize ear traits. E1: Urbana, E2: Aurora, E3: Clayton, E4: Homestead. *P*-values obtained from multiple pairwise-comparisons between the means of groups (locations) using Tukey Honest Significant Differences (Tukey HSD) were given in (**A**)-(**D**). Red points in (**E**) are the experimental sites, where the NAM population was cultivated

Location-specific distributions of maize ear traits are plotted to show the phenotypic differences of plant lines cultivated in different geographical locations. The phenotypic distributions of ear traits were found highly diverse across multiple locations (Fig. 1 A-D). In comparison to the other three places, Urbana, Aurora, and Clayton, all of the four ear traits in Homestead had lower phenotypic means. The phenotypic means in two places, Aurora and Clayton, were almost similar. In addition, phenotypic means in Urbana were larger than phenotypic means in Homestead but smaller than phenotypic means in Aurora and Clayton. For example, phenotypic means of ear weight were 71.17, 79.38, 78.44, and 48.15 for Urbana, Aurora, Clayton, and Homestead, respectively. Three locations, Urbana, Aurora, and Clayton, are relatively closer, while Homestead is much further away (Fig. 1E).

Geographical locations had a substantial impact on the ultimate productions (Fig. 1A-D). The estimated heritability showed that gene × environment interaction effects intensively controlled phenotypic variations of the traits (Fig. 1F). Therefore, gene × environment interactions could be a major factor leading to phenotypic differences in plant line in different locations.

### Estimated heritability for ear traits

The heritability of additive, dominance, epistasis, and gene × environmental interaction effects were calculated and tabulated for the four ear traits (Table 1). The total heritability was estimated to be 0.3731 to 0.6234. Among these traits, the total heritability of ear row number was the largest (${h}_T^2\stackrel{\wedge}{=}$ 0.6234) and the total heritability of ear weight was the smallest (${h}_T^2\stackrel{\wedge}{=}$ 0.3731). Association analyses revealed significant impacts of dominance, dominance-related epistasis, and environmental interaction effects on heritability. Epistasis effects had large impacts on the phenotypic variations of three ear traits, length, rank number, and row number (Fig. 1F). Dominance, dominance-related epistasis, and dominance-related environmental interaction effects largely contributed to phenotypic variations (0.1406 ~ 0.4928), as compared with additive, additive related epistasis, and additive related environmental interaction effects. Heritability due to dominance effects was large for ear length (0.1245) and rank number (0.1007), while heritability due to dominance × dominance epistasis effects was large for ear row number (0.2380) and rank number (0.2023). For ear length, rank number, and row number, heritability attributable to additive and additive × additive epistasis effects was 0.1157, 0.0726, and 0.1041, respectively. It suggests that heterozygous genotypes play very important role for phenotypic performance of ear length, rank number, and row number. Estimated dominance × additive epistasis heritability was large for ear length (${h}_{DA}^2\stackrel{\wedge}{=}$ 0.1418).

**Table 1:** Estimated heritability (%) of genetic effects for ear traits of maize

Trait	${h}_A^2$	${h}_D^2$	${h}_{AA}^2$	${h}_{AD}^2$	${h}_{DA}^2$	${h}_{DD}^2$	${h}_{AE}^2$	${h}_{DE}^2$	${h}_{AAE}^2$	${h}_{ADE}^2$	${h}_{DAE}^2$	${h}_T^2$	${h}_{D+}^2$	${h}_{GE}^2$
**Weight**	11.6	9.43	—	—	—	—	11.65	4.63	—	—	—	37.31	14.06	16.28
**Length**	9.91	12.45	1.66	—	14.18	6.69	10.32	1.08	0.34	—	—	56.63	34.40	11.74
**Rank Number**	6.90	10.07	0.36	—	2.27	20.23	6.63	1.79	0.47	3.50	—	52.22	37.86	12.39
**Row Number**	9.82	8.71	0.59	1.56	7.47	23.8	2.65	1.18	—	—	6.56	62.34	49.28	10.39

${h}_A^2=$ heritability of additive effects; ${h}_D^2=$ heritability of dominance effects; ${h}_{AA}^2=$ heritability of additive × additive epistasis; ${h}_{AD}^2=$ heritability of additive × dominance epistasis; ${h}_{DA}^2=$ heritability of dominance × additive epistasis; ${h}_{DD}^2=$ heritability of dominance × dominance epistasis; ${h}_{AE}^2=$ heritability of additive × environment interaction effects; ${h}_{DE}^2=$ heritability of dominance × environment interaction effects; ${h}_{AAE}^2=$ heritability of additive-additive epistasis × environment interaction effects; ${h}_{ADE}^2=$ heritability of additive-dominance epistasis × environment interaction effects, ${h}_{DAE}^2=$ heritability of dominance-additive epistasis × environment interaction effects, ${h}_T^2=$ total heritability; ${h}_{D+}^2=$ sum of dominance related heritability. ${h}_{GE}^2=$ sum of gene × environment interactions related heritability

Heritability due to gene by environment interaction effects (${h}_{GE}^2$) was comparatively large for four ear traits (0.1628 for weight, 0.1174 for length, 0.1239 for row number, and 0.1039 for rank number) suggesting that genetic effects of several SNPs could be very sensitive to different geographical locations. Ear weight and length had high heritability due to additive × environment interaction effects (0.1165 and 0.1032, respectively), demonstrating that these factors could largely influence phenotypic variations. Heritability due to dominance × environment interaction effects was also found to be relatively large for ear weight, and heritability due to additive × dominance × environment interaction effects was large only for ear row number. Moreover, heritability due to dominance by environment interaction effects had a large influence on the phenotypic variation of these traits. Therefore, estimating gene-environment interaction effects are necessary to understand the complexity of the genetic architecture of ear traits.

### Identified SNPs with no gene × environment interactions

**Fig. 2:**
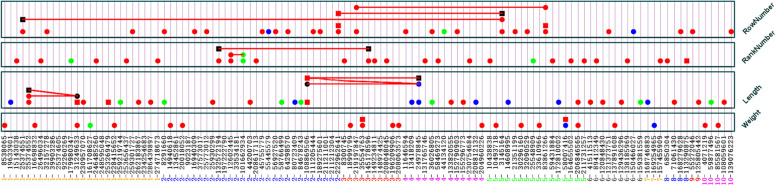
Genetic architecture of maize ear traits. The bottom axis is the SNP IDs. Three shapes, circle, square, and line, indicate additive, dominance and epistasis effects, respectively, while four colors, red, green, blue and black, indicate esistence of main effects only, location-specific effect only, both effects and no both effects, respectively. The different combination of shape and color indicate diffent condition on effect type and their interaction with location; thus, red circle (square, line): SNP with additive (dominance or epistasis) effects but no location-specific additive (dominance or epistasis) effects; green circle (square, line): SNP without additive (dominance or epistasis) effect but with location-specific additive (dominance or epistasis) effect, blue circle: SNP with both additive (dominance or epistasis) and location-specific additive (dominance or epistasis) effects; black circle (square): SNP without additive (dominance) effects but with epistasis effect

Gene by environment (G × E) interaction measures the variations of SNPs effects across multiple environments or locations. It was observed that G × E interaction effects of several identified SNPs were not highly significant (Fig. 2 and Supplementary Fig. S1, Table 1). Such SNPs will be important for breeding environmentally stable varieties.

Ear weight is an important trait that measures ultimate maize productions. Association analysis using base model (without correcting for population stratification) identified eighteen SNPs with highly significant effects (−*log*_*10*_*P* > 5) associated with ear weight, which had no significant G × E interaction effects. However, eleven of these SNPs were not identified using principal component (pc) adjusted model (Supplementary Tables S1 and S3). Identified SNPs using base model were located on seven chromosomes out of ten chromosomes**.** Among them, seventeen SNPs had additive effects, and one SNP had additive and dominance effects. A SNP S5_10,493,718, located in chromosome 5 at 10493718 bp had the most significant and largest positive additive effect (*a*≙ 1.96, *P* = 1.795 × 10^− 22^), was identified using both base and pc-adjusted model. This SNP is near the protein kinase superfamily gene *Zm00001d013405*, which plays a key role in disease resistance system of plants in tropical maize [19]. Therefore, this gene may be involved in increasing maize production by protecting plants from diseases. A SNP S5_206846565 located in chromosome 5 identified using both base and pc-adjusted model had additive effect (*a*≙ 1.44, *P* = 1.308 × 10^− 12^), which is near a variant of Dof zinc finger protein (DOF2.1) gene *Zm00001d017788*. This gene is involved in many processes during plant growth and comprising at least 14 sub-families or types. Another SNP S3_18999799 also identified using both models, had the largest additive effect (*a*≙ –1.90, *P* = 4.817 × 10^− 21^), which is close to the Ribosomal protein L26 gene *Zm00001d039908* and contributed to 0.0079 of phenotypic variations. Negative additive effect of SNP S5_30296160 of gene *Zm00001d014039* (non-intrinsic ABC protein 4) contributed to 0.0067 of phenotypic variations was only identified using base model due its association with family structure. Similarly, SNP S3_35554763 identified using only base model had highly significant positive additive effect (*a*≙ 1.45, *P* = 5.577 × 10^− 13^) and negative dominance effect (*d*≙ –4.62, *P* = 1.485 × 10^− 6^). This variant contributed to 0.0046 of phenotypic variation by homozygous genotypes and 0.0234 of phenotypic variation by heterozygote genotype, and is near the topless-related protein gene *Zm00001d040279*. Most of the base model identified SNPs (12 out of 18 SNPs) are located on three chromosomes (1, 3, and 5). PC-adjusted model exclusively identified 3 SNPs (S1_259223303, S4_234419646, and S8_73905364) which had no significant G × E interaction effects (Supplementary Table S3).

For ear length**,** seventeen SNPs were found on eight chromosomes with highly significant (−*log*_*10*_*P* > 5) genetic main effects using base model, and eleven of the SNPs were not identified using pc-adjusted model. Six SNPs were detected on chromosome 1 using base model, but five of the SNPs were not identified using pc-adjusted model. Fifteen SNPs had highly significant additive effects and two SNPs had dominance effects. Fifteen SNPs with highly significant additive effects were detected which explained approximately 0.2249 of phenotypic variations. Therefore, larger portions of estimated heritability was due to highly significant additive effects, including negative additive effects of five SNPs (${h}_{A-}^2\stackrel{\wedge}{=}$ 0.08.31) and positive additive effects of ten SNPs (${h}_{A+}^2\stackrel{\wedge}{=}$ 0.1418). SNP S10_108059601, variant near the protein XRI1 gene *Zm00001d025182*, was identified using both base and pc-adjusted model, had the largest positive additive effect (*a*≙ 2.62, *P* = 4.340 × 10^− 51^) that contributed to 0.0130 of phenotypic variations. Protein XRI1 of this gene involved in male and female meiotic nuclear division (Uniport: B4FGL0). Meiotic nuclear division refers to a cell cycle process by which the cell nucleus divides as part of a meiotic cell cycle in male or female germ line. SNP S1_25374552 was identified using only base model, which had most significant and largest negative additive effect (*a*≙ –3.07, *P* = 1.746 × 10^− 68^), which accounted for around 0.0179 of phenotypic variations. This SNP is near FRIGIDA-like protein 1 gene *Zm00001d028173*. FRIGIDA-like protein 1 gene controls the regulation of flowering time. It is known to increase RNA levels of flowering locus C. Allelic variation at the FRIGIDA locus is a major determinant of natural variation in flowering time [20]. Base model association analysis identified two SNPs (S1_31249633 and S1_253269479) with highly significant dominance effects, whereas the proportions of variation due to their heterozygous genotypes were 0.0084 ~ 0.0132. SNP S1_253269479 had highly significant positive dominance effects (*d*≙ 2.97, *P* = 4.958 × 10^− 6^) which is a variant near the mitochondrial import receptor subunit TOM5 homolog gene *Zm00001d033186*. SNP S1_31249633, a variant near gene *Zm00001d028339,* had highly significant negative dominance effect (*d*≙ –3.73, *P* = 5.357 × 10^− 6^). Two pairs of epistasis interactions were identified using base model, and estimated effects of the epistasis were relatively large as compared with the main effects. Highly significant positive additive × additive (*aa*≙ 1.64, *P* = 2.60 × 10^− 20^) and dominance × additive (*da*≙ 6.25, *P* = 9.190 × 10^− 16^) epistasis interaction effects were identified between the variants S1_25374552 of gene *Zm00001d028173* and S1_31249633 of gene *Zm00001d028339*. Another epistasis pair, S2_108869506 of gene *Zm00001d004396* and S4_24978845 of gene *Zm00001d049295,* were identified using base model with highly significant positive additive × additive (*aa*≙ 1.30, *P* = 4.724 × 10^− 13^), negative dominance × additive (*da*≙ –5.96, *P* = 1.737 × 10^− 22^), and dominance × dominance (*dd*≙ –7.21, *P* = 2.438 × 10^− 6^) interactions effects. These two epistasis pairs explained 0.0844 and 0.1234 of phenotypic variations, respectively. A number of individual SNPs were identified to contribute small effects but highly significantly identified epistasis SNPs had relatively large effects on ear length, while dominance and dominance related epistasis interactions were revealed as important contributors to phenotypic variations of this trait.

Similar results were found for another important trait of maize, Ear rank number. Base model association analysis identified twenty-four SNPs with highly significant genetic main effects (−*log*_*10*_*P* > 5) for ear rank number, including twenty-four SNPs with individual genetic effects and two pairs of SNPs with epistasis interactions. Among the highly significantly base model identified SNPs, twenty-three SNPs had additive effects (positive for 13 SNPs and negative for 10 SNPs), one SNP had only positive dominance effect and two SNPs had negative epistasis interaction effect. Among the SNPs fifteen individual and two pairs of SNPs were not identified using pc-adjusted model, suggest their associated with family structure. SNP S2_208617113 was identified using both base and pc-adjusted model, which is the variant of UDP-glycosyltransferase 71B1 gene *Zm00001d006450,* had highly significant positive additive effect (*a*≙ 0.3615, *P* = 4.41 × 10^− 15^) that could explain 0.0042 phenotypic variations of rank number (Supplementary Table S1). UDP-glycosyltransferases catalyze transfers the sugar moiety from the uridine-diphosphate activated monosaccharide molecule to the specific acceptor and plays various functions in plant cells, such as high-energy donors, or signaling molecules, and are involved in biosynthesis of cell walls [21]. SNP S5_59431884 was identified using only base model, which is the variant of AIG2-like protein gene *Zm00001d014692* was identified with highly significant negative additive effect (*a*≙ –0.3678, *P* = 5.51 × 10^− 16^), that could explain 0.0044 of phenotypic variations of rank number. AIG2 differentiate between resistance responses mediated by the RPS2 and RPM1 disease resistance genes [22].

Two pairs of epistasis SNPs were identified using base model, in which one pair had highly significant additive × additive epistasis effect and another pair had dominance × dominance epistasis effect. Epistasis SNPs S2_132572194 and S3_145178596, the variants of F-box protein interaction domain containing protein gene *Zm00001d004708* and the unknown gene *Zm00001d041953* had highly significant negative dominance × dominance epistasis effect (*dd*≙ –3.5378, *P* = 9.34 × 10^− 19^, *h*^2^≙ 0.2023) and accounted for 0.2023 of phenotypic variations. This epistasis SNPs could dramatically decrease ear rank number by their heterozygous genotypes. Another base model identified epistasis pair S2_12602445 × S2_101662934 had highly significant negative additive × additive (*aa*) epistasis effect (*aa*≙ –0.2348, *P* = 6.953 × 10^− 7^). The genetic mechanism of ear rank number is similar to the genetic mechanism of ear length, whereas the dominance and dominance related interaction effects are important components of variation of these traits.

For ear row number base model analysis discovered highly significant genetic main effects for twenty-four individual SNPs and three pairs of epistasis SNPs. Thirteen of the SNPs with individual effects were not identified using pc-adjusted model. In base model analysis, a large portion of total heritability was due to dominance and dominance-related epistasis effects (${h}_{D+}^2\stackrel{\wedge}{=}$ 0.4928) for ear row number (Table 1). The genetic effects of individual SNPs were relatively small as compared with epistasis interactions. The dominant related epistasis interactions are recognized to have large effects. The heritability due to heterozygous loci were 0.0113 ~ 0.0259 for the SNPs, which were identified with highly significant dominance effects; while, the heritability due to heterozygous genotypes were 0.0618 ~ 0.2018 for the SNPs, which had dominance related epistasis effects (Table S1). Therefore, only a small portion of individuals had heterozygous genotypes with large impacts due to dominance and dominance-related epistasis effects. SNP S5_21351199, the variant of Tryptophan N-monooxygenase 2 gene *Zm00001d013818*, had most highly significant positive additive effect (*a*≙ 0.2616, *P* = 1.32 × 10^− 49^) and SNP S3_219197847 a variant near the Zn-dependent exopeptidases superfamily protein gene *Zm00001d044102*, had second most highly significant positive additive effect (*a*≙ 0.2042, *P* = 3.76 × 10^− 31^). S5_21351199 was identified using base model only, but SNP S3_219197847 was identified using both models. A SNP S8_168279628 identified using both models, had largest highly significant negative additive effect (*a*≙ –0.2052, *P* = 1.4 × 10^− 31^), which is the variant of E3 ubiquitin-protein ligase RGLG1 gene *Zm00001d012101*. Two SNPs (S2_222962641 and S5_83861266) identified using both models, had highly significant additive and dominance effects; whereas, SNP S2_222962641, a variant near the heavy metal transport/detoxification superfamily protein gene *Zm00001d007130*, had negative additive and dominance effects (*a*≙ –0.1606, *P* = 4.832 × 10^− 20^ and *d*≙ –0.5609, *P* = 5.88 × 10^− 9^); whereas, the dominance effects of this SNP could explain 2.59% phenotypic variations and ear row number could be decreased by heterozygous genotype. SNP S5_83861266, the variant of probable arabinose 5-phosphate isomerase gene *Zm00001d015306*, had positive additive and dominance effects (*a*≙ 0.2263, *P* = 1.452 × 10^− 37^ and *d*≙ 0.3703, *P* = 3.864 × 10^− 6^); whereas, the dominance effects of this SNP could explain 0.0113 of phenotypic variations and its heterozygous genotype could increase row number. Among three base model identified epistasis pairs, two pairs had dominance-related epistasis effects. Epistasis of S2_222962641 × S5_3145164 was identified with highly significant positive dominance × dominance epistasis effect (*dd*≙ 1.5657, *P* = 4.986 × 10^− 16^), which are variants near the heavy metal transport/detoxification superfamily protein genes *Zm00001d007130* and DNA gyrase subunit A chloroplastic/mitochondrial gene *Zm00001d013006*, respectively. Another epistasis of S1_25374551 × S5_3145164 was identified with highly significant positive dominance × additive epistasis effect (*da*≙ 0.6127, *P* = 1.339 × 10^− 16^), which are variants near the FRIGIDA-like protein 1 gene *Zm00001d028173* and DNA gyrase subunit A chloroplastic/mitochondrial gene *Zm00001d013006*, respectively. Epistasis of S3_219197847 × S5_83861266 was identified with highly significant negative additive × additive epistasis effect (*aa*≙–0.1177, *P* = 5.151 × 10^− 11^). These epistasis SNPs are variants near genes *Zm00001d044102* (Heavy metal transport/detoxification superfamily protein) and *Zm00001d015306* (Probable arabinose 5-phosphate isomerase), respectively. Several individual SNPs were identified with small effects, but several pairs of epistasis interactions had large effects on the number of ear rows in the maize NAM population, while dominant and dominant related epistasis interactions were revealed as important contributors to phenotypic variations.

### Identified SNPs with highly significant gene × environment interaction

Location-specific genetic effects were discovered as important variants for four maize ear traits. It was observed that twenty-four individual SNPs had highly significant (−*log*_*10*_*P* > 5) location specific genetic effects for four ear traits using base models (Fig. 2, Supplementary Table S2). Three base model identified SNPs had highly significant G × E (−*log*_*10*_*P* > 5) effects associated with ear weight (Table S2). SNP S5_58007416, had highly significant positive additive (*a*≙ 3.47, *P* = 3.043 × 10^− 64^) and dominance effects (*d*≙ 3.72, *P* = 7.279 × 10^− 7^). Additive effect was smaller in location Aurora (*ae*_2_≙ –2.65, *P* = 9.733 × 10^− 11^) and larger in location Clayton (*ae*_3_≙ 2.57, *P* = 8.135 × 10^− 11^). This SNP is the variant near the protein chloroplast import apparatus 2 gene *Zm00001d014664* that contributed to 0.0263, 0.0151, and 0.0149 of phenotypic variations by additive effect, dominance effect, and additive × environmental epistasis effects, respectively, in two locations (Aurora and Clayton). However, effects of the SNPs were not significantly identified using pc-adjusted model. SNP S7_169254965, the variant of LRR receptor-like serine/threonine-protein kinase RPK2 gene *Zm00001d022066*, had a highly significant negative additive effect (*a*≙ –0.95, *P* = 2.895 × 10^− 6^). However, the additive effect of this SNP largely varied across locations, minimum in Aurora (*a* + *ae*_2_≙ –4.18) and maximum in Clayton (*a* + *ae*_3_≙ 0.97). A leucine-rich repeat (LRR) is a protein structural motif that forms an α/β horseshoe fold which composed of repeating 20 ~ 30 amino acid stretches that are unusually rich in the hydrophobic amino acid leucine [23]. This SNP was also identified using pc-adjusted model. SNP S1_161708627, the variant of cycloartenol synthase gene *Zm00001d030813,* had only negative additive × environmental interaction effect (*ae*_1_≙ –2.99, *P* = 4.887 × 10^− 14^) in the Urbana. Gene *Zm00001d030813* that is a plant enzyme that catalyzes the cyclization of (S)-2,3-epoxysqualene to cycloartenol [24]. This SNP was not identified using pc-adjusted model.

Base model association analysis identified fifteen SNPs with highly significant location specific genetic effects for ear length (Table S2), however, nine of them were not identified using pc-adjusted model. SNP S7_168746883 had highly significant additive effect (*a*≙ 1.71, *P* = 1.716 × 10^− 23^), and additive by environmental interaction effect in the locations Urbana, Aurora, and Clayton (*ae*_1_≙ 2.67, *P* = 1.426 × 10^− 14^; *ae*_2_≙ –1.81, *P* = 4.081 × 10^− 7^; and *ae*_4_≙ –1.58, *P* = 2.476 × 10^− 6^) that accounted for 0.0119 of phenotypic variations. This SNP is near the SNF2 domain-containing protein gene *Zm00001d022046*. The SNF2 domain-containing proteins associated with ear length that includes transcriptional regulation, chromosome stability during mitosis, and various aspects of processing of DNA damage, including nucleotide excision repair, recombination pathways and post-replication daughter strand gap repair [25]. This SNP also identified using pc-adjusted model. Variants of four genes *Zm00001d053008*, *Zm00001d013575*, and *Zm00001d049034* had larger positive additive effects in the location Urbana (*a* + *ae*_1_≙ 3.93, 2.58, and 1.49 respectively), identified using both models. Seven SNPs had only location specific additive effects (S1_259219744, S2_8291660, S2_58766499, S2_84678243, S4_36026805, S6_159381559 and S10_59877496), indicating that these SNPs interact with environment to control ear length. Effects of these SNPs were not identified using pc-adjusted model.

For ear rank number, there were only three Aurora specific additive effect of SNPs identified using base model (Table S2), two of them were not significant in pc-adjusted model. SNP S2_101662934, the variant of E3 ubiquitin-protein ligase MBR2 gene *Zm00001d004300*, had positive additive × environment interactions effect (*ae*_2_≙ 0.42, *P* = 3.411 × 10^− 6^). Various expression patterns indicated that this gene play crucial roles in the response of plants to stress and involved in various physiological and developmental processes in maize [26]. This SNP was not identified using pc-adjusted model. SNP S1_179980347, the variant of pathogenesis-related protein 5 gene *Zm00001d031158* was identified using both model, but S5_25548909, the variant of unknown gene *Zm00001d013944*, was identified using only base model.

For ear row number, three SNPs (S2_55445579, S6_150466027, and S4_44134120) were identified with highly significant environment-specific additive effects using base model, whereas S4_44134120 was not identified using pc-adjusted model. SNP S2_55445579, near the variant of unknown gene *Zm00001d003706*, had negative additive effect (*a*≙ –0.08, *P* = 6.258 × 10^− 6^). However, additive effects of this SNP largely varied across environments, maximum in location Urbana (*a* + *ae*_1_≙ 0.112) and minimum in location Aurora (*a* + *ae*_2_≙ –0.249). SNP S6_150466027, near the variant of Ubiquitin-associated/translation elongation factor EF1B protein gene *Zm00001d038171*, had highly significant negative additive and positive additive by environment interaction effects (*a*≙ –0.112, *P* = 1.432 × 10^− 10^ and *ae*_4_≙ 0.1862, *P* = 2.925 × 10^− 8^). Again, SNP S4_44134120, the variant of BTB/POZ and MATH domain-containing protein 1 gene *Zm00001d049774*, had only a highly significant environment-specific positive additive effects (*ae*_4_≙ 0.302, *P* = 2.304 × 10^− 18^).

**Fig. 3:**
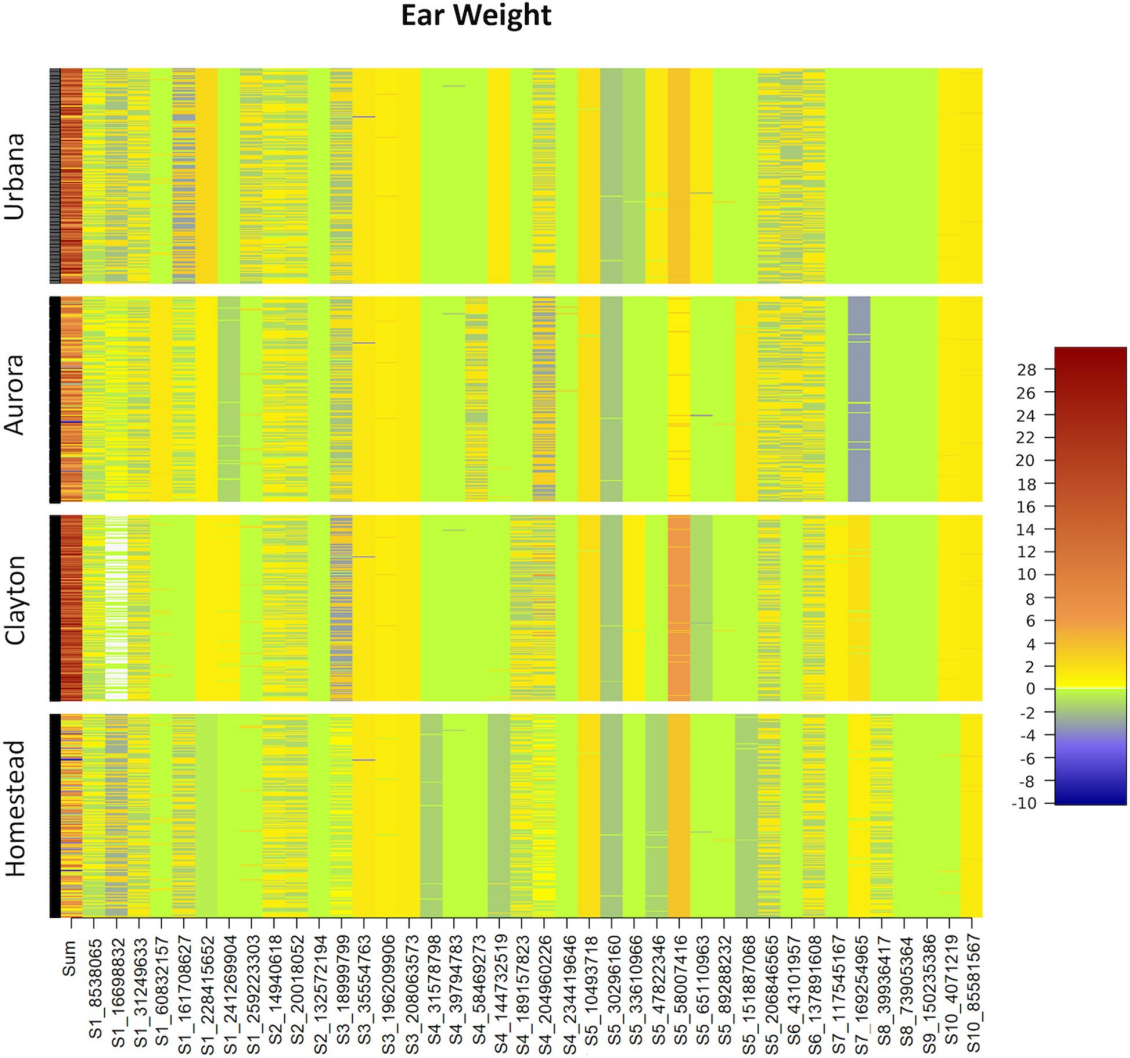
Heat map for genetic effects of the ear weight associated SNPs in the four different locations. This figure demonstrate that genetic effects of a SNP could be varied in different geographical locations. First column is for sum of the genetic effects of all the identified SNPs. Color legend is given in the right side of the figure

We plotted location specific SNP effects associated with ear weight to demonstrate gene × environmental interaction effects (Fig. 3). It was observed that total genetic effects of the lines were relatively higher for Urbana, Aurora, and Clayton as compared to Homestead. Moreover, SNP effects largely varied among different locations.

### Annotation and function prediction of candidate genes

It was observed that some candidate genes are members of well-known gene families that have vital functions in plant life. SNP S2_14940618 is the variant near the Probable L-type lectin-domain containing receptor kinase S.5 gene *Zm00001d002536*. The lectin-domain is connected in plant growth, development and stress tolerance [27]. SNP S1_262183867 is the variant of transcription factor bHLH28 of gene *Zm00001d033407*. The bHLH28 transcription factor proteins play controlling roles in upliftment B73 reference genome in maize [28]. We collected the GO terms corresponding to the identified genes from Maize GAMER database [29]. We drew a tree map of biological process of the genes using R-script generated from Revigo (Supplementary Fig. S1). It was observed that large number of GO terms were reduced to the only several numbers of GO terms, including cell development, cytokine metabolisms, response to chemical, etc. Cell development is comprised of many others GO terms including pollen development, xylem development, seed germination, positive regulation of flower, leaf senescence, cell growth, female meiotic division etc. A large set of genes are involved in cytokine metabolism and play important roles in various physiological functions in the plant [30]. A group of genes are response to chemicals. Some genes are response to salt stress, oxidative stress, cold acclimation, bacterium, freezing, UV-B, water deprivation, organic substance, carbohydrate, ethylene, detection biotic stimulus etc. Effects of these genes could be varied in different geographical locations due to different stress conditions. We performed gene expression analysis of the candidate genes facilitated by maize inflorescence database (Supplementary Fig. S2) [31]. We chose tip of the ear (ear_tip), middle of the ear (ear_mid), base of the ear (ear_base), tassel at stage 1 (tassel_stg1), tassel at stage 2 (tassel_stg2), and tassel at stage 3 (tassel_stg3) from experiments in ear and tassel development series; wild type ear at 2 mm (wt_2mm_ear) from experiments in fascinated ear (fea4); and wild type ear 2–5 mm from experiments in knotted1. Analysis results showed that many of the identified genes highly express in ear and tassel. Therefore, it can be concluded that the identified genes might play important role in controlling ear traits.

### SNPs associated with multiple ear traits

Some identified SNP had significant effects on at least two maize ear traits, as shown in Table 2. Four SNPs were associated with two of three ear traits weight, length, and row number. Of these sets of SNPs, SNP S2_8291660, the variant of F-box only protein 7 gene *Zm00001d002211*, had negative additive effect on ear row number (*a*≙ –0.14, *P* = 3.047 × 10^− 16^) and location Urbana specific negative additive effect (*ae*_1_≙ –1.754, *P* = 3.133 × 10^− 7^) on ear length. Identified F-box proteins are involved in many plant vegetative growth and reproductive development. For example, F-box protein-FOA1 involved in abscisic acid (ABA) signaling to affect the seed germination [32]. ACRE189/ACIF1 can regulate cell death and defense when the pathogen is recognized in the Tobacco and Tomato plant [33]. SNP S4_36026805, the variant of myb-like transcription factor family protein gene *Zm00001d049581*, had positive additive effect (*a*≙ 0.13) for ear row number and location Homestead specific negative additive effect (*ae*_4_≙ –2.001) for ear length.

**Table 2:** Identified SNPs associated with multiple ear traits

SNPs	Gene	Traits	Gene Descriptions
S2_8291660	*Zm00001d002211*	Row, Length	F-box only protein 7
S4_36026805	*Zm00001d049581*	Row, Length	myb-like transcription factor family protein
S1_31249633	*Zm00001d028339*	Weight, Length	Whole genome shotgun sequence of line PN40024 scaffold_7.assembly12x (Fragment)
S5_206846565	*Zm00001d017788*	Weight, Length	Dof zinc finger protein DOF2.1

Another SNP S1_31249633, variant of gene *Zm00001d028339,* had positive additive effect (*a*≙ 1.33, −*log*_*10*_*P* = 4.580 × 10^− 11^) on ear weight but negative dominance effect (*d*≙ –3.73, −*log*_*10*_*P* = 5.357 × 10^− 6^) on ear length, indicating that dominance effects of this gene decreased ear length. SNP S5_206846565 of Dof zinc finger protein DOF2.1 gene *Zm00001d017788* had positive additive effects both for ear weight and length (*a*≙ 1.44, 1.90; −*log*_*10*_*P* = 1.308 × 10^− 12^, 8.219 × 10^− 28^, respectively), showing that this gene *Zm00001d017788* could simultaneously increase ear weight and length.

### Breeding potential of predicted lines

To further improve corn production, genetic information obtained from association studies could be used to design superior lines (SL) and superior hybrids (SH), listed in Table 3 and Supplementary Table S4. Based on the predicted superior genotypes, the total genetic effects of individual line can be estimated. The predicted total genotypic values of hybrid lines with only heterozygous genotypes in all the identified SNP (*Qq*) were positive for four traits. The predicted genotypic value of heterozygotes (*Qq*) combination was larger than that of homozygote (*QQ*) combination for ear weight, rank number, and row number, but smaller for ear length. It is suggested that heterozygous genotypes at these loci may potentially increase the breeding value of ear weight, rank number, and ear row number, but not for ear length. There were slight differences in total genotypic value between the best line and the predicted superior line for four traits. However, the total genetic effects of location-specific best lines and the predicted superior lines differ greatly for two traits (weight and length). Based on genetic effects of the identified SNPs, it can be suggested that pure-line breeding may only have limited potential to improve ear rank number and row number over current best lines of NAM population. For ear weight and length, there are large differences in total genotypic value between the best line and the predicted superior hybrid line. Therefore, it can be suggested that breeding with combination of homozygous and heterozygous genotypes may have larger potential to improve ear traits.

**Table 3:** Prediction of the total genotypic values of four ear traits in maize

Entry	G	G + GE1	G + GE2	G + GE3	G + GE4
**Weight**	μ = 68.88	μ = 70.94	μ = 78.07	μ = 78.41	μ = 48.11
***QQ***	10.54	− 0.67	10.50	17.89	14.19
***qq***	−10.54	0.67	−10.50	− 17.89	−14.19
**F**_**1**_	13.71	13.71	14.56	17.37	10.74
**Best Line (+)**	25.55 (Z004E0030)	31.18 (Z022E0143)	32.81 (Z016E0049)	37.60 (Z014E0113)	27.97 (Z012E0108)
**Superior Line (+)**	26.15	39.95	36.44	36.10	34.41
**Superior Hybrid (+)**	39.18	50.16	53.29	53.43	46.45
**Length**	μ = 126.73	μ = 128.64	μ = 138.62	μ = 139.42	μ = 100.29
***QQ***	11.13	26.10	−0.795	14.97	−1.67
***qq***	−5.25	−21.12	6.67	−9.094	7.55
**F**_**1**_	1.39	1.39	1.39	0.77	1.39
**Best Line (+)**	27.10 (Z006E0233)	48.18 (Z007E0007)	26.42 (Z006E0150)	28.43 (Z003E0140)	24.44 (Z010E0164)
**Superior Line (+)**	27.75	54.92	33.76	33.83	32.83
**Superior Hybrid (+)**	40.94	63.80	58.41	49.07	47.29
**Rank Number**	μ = 22.63	μ = 21.65	μ = 24.98	μ = 24.92	μ = 18.97
***QQ***	0.34	−1.24	0.67	0.03	0.70
***qq***	−0.34	1.24	−0.67	−0.03	−0.71
**F**_**1**_	1.72	1.72	1.72	0.25	1.72
**Best Line (+)**	5.43 (Z016E0164)	6.08 (Z016E0164)	6.45 (Z012E0179)	5.74 (Z016E0164)	6.75 (Z016E0164)
**Superior Line (+)**	4.80	7.67	7.65	5.12	7.05
**Superior Hybrid (+)**	10.68	12.53	13.06	9.80	13.11
**Row Number**	μ = 14.56	μ = 14.22	μ = 15.29	μ = 14.80	μ = 13.94
**QQ**	−0.48	−0.38	−0.65	−0.35	−0.54
**qq**	0.24	0.15	0.41	0.12	0.31
**F**_**1**_	1.05	1.05	1.05	1.05	0.67
**Best Line (+)**	2.25 (Z019E0115)	2.67 (Z019E0158)	2.42 (Z019E0115)	2.31 (Z026E0166)	2.11 (Z026E0166)
**Superior Line (+)**	2.82	3.72	2.99	2.95	2.78
**Superior Hybrid (+)**	4.31	5.39	4.47	4.43	4.39

Mean, estimated average genotypic value; E1 (Urbana), E2 (Aurora), E3 (Clayton), and E4 (Homestead). *QQ*, predicted line with only *QQ* homozygous genotypes of all significant SNPs; *qq*, predicted line with only *qq* homozygous genotypes of all significant SNPs; F_1_, predicted line with only *Qq* heterozygous genotypes; Best line (+), maximum positive genetic value of existing line; Superior line (+), predicted line with maximum positive genetic value by combining homozygous genotypes (*QQ*, *qq*) for all significant SNPs; Superior hybrid (+), predicted line with maximum positive genetic value by combining homozygous (*QQ*, *qq*) and heterozygous (*Qq*) genotype for all significant SNPs

For ear weight, overall total genetic values of the non-B73 allele homozygous (*QQ*) combinations were − 0.67 ~ 17.89 in four locations, with the smallest in Urbana. Therefore, non-B73 allele homozygous (*QQ*) combinations for the identified SNPs could perform poorly in Urbana. The predicted total genetic effect for *F*_1_ hybrid (13.71) was larger than non-B73 allele homozygous (*QQ*) genotypes in all the four locations. Maximum total genetic effects across locations was revealed for line Z004E0030 (25.55) called as the best line (BL) across locations, whereas location specific BLs were Z022E0143 (31.18) in Urbana, Z016E0049 (32.81) in Aurora, Z014E0113 (37.60) in Clayton, and Z012E0108 (27.98) in Homestead. Environment-specific overall genetic effects were large in three locations (Urbana, Aurora, and Clayton) compared to the total genetic effect of Homestead. Comparison between existing line Z004E0030 and superior hybrid (SH) across the identified SNPs showed that genotypes of 9 highly significant SNPs were different to each other, whereas genotypes of two SNPs (S4_204960226 and S5_58007416) had heterozygous genotypes for SH.

For ear length, the overall genetic effects of the non-B73 allele homozygous (*QQ*) combinations were − 1.67 ~ 26.10 across four different locations. Total genetic effects for non-B73 allele homozygous (*QQ*) combinations were negative in Aurora (− 0.795) and Homestead (− 1.67), but positive in Urbana (26.997) and Clayton (14.972). The huge difference was due to the influence of additive × environment interaction. The predicted total genetic effects of *F*_1_ hybrids (1.39) were smaller than that of non-B73 allele homozygous (*QQ*) genotype. Line Z006E0233 (27.20) revealed as the BL across environments, and location-specific BLs were Z007E0007 (48.18) in Urbana, Z006E0150 (26.42) in Aurora, Z003E0140 (28.43) in Clayton, and Z010E0164 (24.44) in Homestead. Environment-specific total genetic effects were large for Urbana, Aurora, and Clayton, compared to total genetic effects in Homestead. Difference between existing line Z006E0233 and SH was due to the genotypes of eighteen highly significant SNPs, whereas genotypes of four SNPs (S1_253269479, S4_208812132, S5_14608995, and S6_129436296) were heterozygote for SH.

For ear rank number, overall total genetic effects of the non-B73 allele homozygous (*QQ*) combinations was 0.34, which varied − 1.24 ~ 0.71 in four different locations. The predicted total genetic effect of *F*_1_ hybrid (1.72) was larger than that of non-B73 allele homozygous (*QQ*) genotype. The line Z016E0164 (5.43) revealed as BL across environments, whereas environment specific BLs were Z016E0164 in three locations (6.08 in Urbana, 5.74 in Clayton, and 6.75 in Homestead) and Z012E0179 (6.45) in location Aurora. Location-specific total genetic effects were large for Aurora and Clayton and smaller for Urbana and Homestead. The predicted superior positive line [superior line (+)] could provide insight for crop improvement along with the optimum homozygous genotypes (*QQ*, *qq*) combination. Total genetic effect of the predicted SL had ear rank number 4.80, which is mostly like the existing BL (Z016E0164), suggesting that using only homozygous genotypes combination would have very little scope for further improvement. Difference between existing line Z016E0164 and SH was due to the genotypes of nine highly significant SNPs, whereas genotypes of four SNPs (S3_145178596, S5_164655502, S6_89412349, and S8_113290875) had heterozygote for SH. For ear row number, the total genetic effect of the non-B73 allele homozygous (*QQ*) combinations was − 0.48, which varied − 0.38 ~ − 0.65 in four different locations. For ear row number existing line Z019E0115 revealed as the BL across environments and in Aurora. Z026E0166 line identified as BL in two locations, Clayton and Homestead, and Z019E0158 identified as BL in Urbana. Total genetic effects were large for Urbana, Aurora, and Clayton but smaller in Homestead. As similar to other ear traits, the total genetic effect of the SH was larger than existing lines for ear rank number, indicating that further improvement is possible with the optimum homozygous and heterozygous combinations.

## Discussions

Maize (*Zea mays*) is one of the important cereal crops. Since the release of the B73 reference genome, many GWASs of complex traits of maize have been performed to discover the genetic mechanism of complex traits for further breeding improvement [3, 14, 34], to meet the ever-increasing demands for corn production. Economically important traits of maize are frequently inherited in a numerical way, where genetic mechanism are functional to the interactions between multiple genes and gene × environment interactions [35]. In this study, two different types of models, base model and principle component adjusted model, were used for trait analysis. Principle component (PC) adjusted models were not detected a subset of the base model identified SNPs due to their association with family structure. Unidentified SNPs in the pc-adjusted model might have true association with phenotypic traits; however, further experimental validation is required. SNPs associated with family structure are the factors for mean differences of phenotypic traits between families. Since, NAM population had very low LD among SNPs; therefore there have more chances that the identified SNPs had true association with traits.

From phenotypic distribution, it was observed that phenotypic means of four ear traits largely varied in four different locations (Fig. 1A-D). Since maize was grown in Urbana, Aurora, and Clayton in summer, and grown in Homestead in winter, season could be a factor for smaller ear traits in this location. Heritability estimation using the full genetic model approach showed a large heritability due to gene × environment interaction effects (Fig. 1F). Consequently, the phenotypic distinction in multiple locations could be held due to gene × environment interaction effects. It was also noticed that phenotypic variances depend on the distances and direction of the locations. For example, Clayton and Urbana positioned in different directions from Aurora. Three locations look like corner points of a triangle on the map (Fig. 1E). Phenotypic distributions were more like Aurora and Clayton than Aurora and Urbana. It could be due to geographical locations and gene-environment interactions.

Similar to our recent study on maize leaf traits [13] and days to silk [14], a large portion of phenotypic variation was estimated for dominance and dominance-related epistasis effects of multiple SNPs for four ear traits (${h}_{D+}^2\stackrel{\wedge}{=}$ 0.1406 for weight, 0.3440 for length, 0.3786 for rank number, and 0.4928 for row number). However, many dominance and dominance-related epistasis effects were not highly significant, that could be due to the lower frequency of the heterozygote genotypes in the NAM population.

The previous study of maize ear traits with different experimental cross identified 17 ~ 34 SNPs [8]. Similar numbers of individual SNPs were identified in this study. Moreover, this study identified several locations of specific SNPs and pairs of epistasis SNPs. There are 21 ~ 34 highly significant SNPs with genetic main effects, epistasis, and environmental interaction effects identified for four ear traits. It was observed that G × E effects were not highly significant for 17 ~ 24 SNPs. Selecting individual lines for the breeding program based on these stable effects of SNPs may provide similar yields in multiple locations for traits of interest.

However, if breeders expect higher productions at specific locations, they also need to consider SNPs with highly significant G × E effects. From Supplementary Table S2, it was observed that for each of the three traits (ear weight, rank number, and row number) only three SNPs had highly significant G × E effects. Therefore, ignoring these SNPs may not largely influence the expected outcomes for these traits. However, for ear length, fifteen SNPs had highly significant G × E effects. Therefore, ignoring the G × E effects of these fifteen SNPs may produce unexpected average ear lengths at multiple locations. Although the ear traits are phenotypically correlated, only four SNPs were identified associated with multiple traits. Therefore, improving maize ear needed to consider all the SNPs which are associated with different ear traits.

Genetic effects estimated from the association analysis were used to predict SHs and observed a large scope of further improvements by optimum homozygote and heterozygote combination. However, in order to improve each ear trait, it is necessary to modify the genotypes of several SNPs. As compared to the current best lines predicted superior hybrids were different for 9 ~ 20 SNPs.

The G × E interaction effect is revealed as one of the major causes for the difference in phenotypes at multiple locations. Estimated breeding values for ear traits suggest, there has a large scope for further improving the maize ear traits. This study reflects that, in order to improve the maize ear, it is necessary to consider the additive, dominance, epistasis, and G × E interaction effects of the associated SNPs. RNAseq analyses revealed that identified SNPs might play important role in controlling ear traits. Moreover, it was observed that different genotypes combinations of multiple SNPs could be better for different environments. We have tabulated the multi-locus genotypes for optimum production based genetic materials of NAM population in different locations. Breeders may consider this information for improving future lines.

## Conclusions

Use of appropriate statistical genetic model play a crucial role in robust identification of associated SNPs for complex trait association analysis, which is the fundamental to enriching knowledge about genetic architecture. We employed full genetic model approach considering different mode of genetic effects and environmental interactions to explore underlying genetic architecture of Maize ear traits to facilitate marker assisted breeding. This study suggests different genotype combinations in different geographical regions may have a greater potential to improve Maize ear traits.

## Methods

### Genotype and phenotype data

The genotype (ZeaGBSv2.3) data was downloaded through CyVerse Discovery Environment (Data Store file path*: /iplant/home/shared/panzea/genotypes/GBS/v23/) and phenotypic data was downloaded from https://www.panzea.org/phenotypes. Maize ear traits from the NAM population were investigated in this study. This population was cultivated in the United States, which consists of 5000 lines from 25 families, developed by crossing 25 different inbred lines with the B73 reference line, and then self-pollinated [34, 36]. Ear traits collected in four locations (Urbana, Aurora, Clayton, and Homestead) in 2006 were analyzed by the software of *QTXNetwork* [16]. Maize was grown in Urbana, Aurora, and Clayton in summer (planting dates: 08-05-2006, 09-05-2006, and 01-05-2006, respectively), and grown in Homestead in winter (planting dates: 22-09-2006). Therefore, both geographical location and season could be factors that influence phenotypic traits. In this study, geographical locations and seasons were treated as environmental factors. Total non-missing phenotypic observations were 14,811 for ear weight, 15,932 for ear length, 14,972 for ear rank number, and 15,441 for ear row number. The quality of genotype and phenotype data was checked and the outliers of phenotype data were removed. Initially we removed phenotypic outliers based on quartile and interquartile range. Phenotypic data larger than Q3 + 1.5 × IQR and smaller than Q1–1.5 × IQR were removed, where Q1 is the first quartile, Q3 is the third quartile, and IQR is the inter-quartile range. The phenotypes were also filtered according to a distribution-based outlier detection of residuals (∣*ε* − *μ*_*ε*_ ∣ /*σ*_*ε*_ > 3). SNPs with MAF < 0.05 and call rate < 90% were discarded.

### Statistical methods and analysis

Statistical analysis was implemented with *QTXNetwork* for dissecting genetic architecture of *maize* ear traits. Generalized multi-factor dimensionality reduction (GMDR) method was used to scan 472,469 SNPs by one dimensional (1D) for main effects, two dimensional (2D) and three dimensional (3D) for epistasis interactions using the GMDR-GPU module of *QTXNetwork* [37]. SNPs detected after GMDR filtering included: 767 for ear length, 713 for ear rank number, 777 for ear row number, and 729 for ear weight. Then, QTS module of *QTXNetwork* was used for association analysis on the detected SNPs. Genetic main effects (*a*, *d*) and epistasis effects (*aa*, *ad*, *da*, *dd*) of SNPs are considered as fixed; environment (*e*) and gene *×* environment interaction effects (*ae*, *de*, *aae*, *ade*, *dae*, *dde*) are considered as random. The phenotypic values of the *k-th* line in the *h-th* location (*y*_*hk*_) can be expressed by the following mixed linear full genetic model:

$${\displaystyle \begin{array}{l}{y}_{hk}=\mu +{c}_{hk}+\sum \limits_i{a}_i{x}_{A_{ik}}+\sum \limits_i{d}_i{x}_{D_{ik}}+\sum \limits_{i<j}{aa}_{ij}{x}_{AA_{ij k}}+\sum \limits_{i<j}{ad}_{ij}{x}_{AD_{ij k}}+\sum \limits_{i<j}{da}_{ij}{x}_{DA_{ij k}}+\sum \limits_{i<j}{dd}_{ij}{x}_{DD_{ij k}}+{e}_h\\ {}\kern1.44em +\sum \limits_i{ae}_{ih}{u}_{AE_{ih k}}+\sum \limits_i{de}_{ih}{u}_{DE_{ih k}}+\sum \limits_{i<j}{aa e}_{ij h}{u}_{{AA E}_{ij h k}}+\sum \limits_{i<j}{ad e}_{ij h}{u}_{{AD E}_{ij h k}}+\sum \limits_{i<j}{da e}_{ij h}{u}_{{DA E}_{ij h k}}+\sum \limits_{i<j}{dd e}_{ij h}{u}_{{DD E}_{ij h k}}+{\varepsilon}_{hk}\\ {}\kern0em \end{array}}$$

where *μ* is the population mean; *c*_*hk*_ is the cofactor of the model, e.g. principal component for adjusting population structure; *a*_*i*_ is the additive effect of the *i*-*th* locus with coefficient ${x}_{A_{ik}}$ (1 for *QQ*, 0 for *Qq,* − 1 for *qq*); *d*_*i*_ is the dominance effect of the *i*-*th* locus with coefficient ${x}_{D_{ik}}$ (1 for *Qq,* 0 for *QQ* and *qq*); *aa*_*ij*_, *ad*_*ij*_, *da*_*ij*_, and *dd*_*ij*_, are the digenic epistasis effects with coefficients ${x}_{AA_{ijk}}$ (1 for *QQ × QQ* and *qq × qq,* − 1 for *QQ × qq* and *qq × QQ*, and 0 for others), ${x}_{AD_{ijk}}$ (1 for *QQ × Qq,* − 1 for *qq × Qq*, and 0 for others), ${x}_{DA_{ijk}}$ (1 for *Qq × QQ,* − 1 for *Qq × qq*, and 0 for others) and ${x}_{DD_{ijk}}$ (1 for *Qq ×Qq*, and 0 for others); *e*_*h*_ is the effect of the *h*-*th* location (1 for Urbana, 2 for Aurora, 3 for Clayton, and 4 for Homestead); *ae*_*ih*_ is the additive × location interaction effect of the *i*-*th* locus in the *h*-*th* location with coefficient ${u}_{AE_{ihk}}$; *de*_*ih*_ is the dominance × location interaction effect of the *i*-*th* locus in the *h*-*th* location with coefficient ${u}_{DE_{ihk}}$; *aae*_*ijh*_, *ade*_*ijh*_, *dae*_*ijh*_, and *dde*_*ijh*_ are the digenic epistasis × location interaction effects in the *h*-*th* location with coefficient ${u}_{AAE_{ijhk}}$, ${u}_{ADE_{ijhk}}$, ${u}_{DAE_{ijhk}}$, and ${u}_{DDE_{ijhk}}$; and *ε*_*hk*_ is the residual effect of the *k*-*th* individual in the *h*-*th* location. Both principal components adjusted and non-adjusted (base) models were analyzed in this study.

Henderson method III [38] was used to construct the F-statistic test for association analysis. Permutation test [39] was conducted by a total of 2000 times for calculating the critical *F*-value to control the experiment-wise type I error (α < 0.05). The SNP effects were estimated by using the MCMC (Markov Chain Monte Carlo) algorithm with 20,000 Gibbs sample iterations [16, 40–42]. The critical experiment-wise *P* value (*P*_*EW*_-value) was thus calculated for controlling the experiment-wise type I error (*P*_*EW*_ < 0.05). More detail about the methodology is available in the supplementary text 1.

### Annotation of candidate genes of identified SNPs

B73 maize genome reference sequence was used to identify putative candidate genes based on SNPs which significantly associated with the four ear traits. Candidate genes corresponding to the maize ear SNPs were collected from the GRAMENE database [43]. The function of candidate genes was collected from the database UniProt [44], where the number of genes collected from the GRAMENE database was kept synchronization. The NCBI gene database and Google’s literature are very useful database for searching identified SNPs and their associated gene functions. As in the previous publication [13], the Pfam website was used for searching the domain name of the candidate genes [45].

### Breeding design using SNP markers

The method designing superior line and the superior hybrid was developed by [46]. This approach can be used in predicting potential of improving plants traits based on SNP-based association analysis. The optimum multi-locus combinations of all the identified SNPs were predicted using Enumeration algorithm and stepwise tuning algorithm.

## Supplementary Information

**Additional file 1: Text S1.** Details about the statistical models used for analyses. **Table S1.** Highly significant SNPs identified by using the base model with no G × E effects for four maize ear traits Effect: *a* = additive effect, *d* = dominance effect,. *aa* = additive × additive interaction effect, *dd* = dominance × dominance interaction effect. SE: standard error of estimated effect/predicted effect. –*Log*_*10*_*P*: minus *log*_*10*_ (*P*-value), *h*^*2*^: estimated heritability. Bold underlined SNPs are strongly associated with family structure and were not identified in the principal component adjusted model. SNPs written in regular word were detected in both base and principal component adjusted model. **Table S2.** Highly significant SNPs identified by using the base model with G × E effects for four maize ear traits. Effect: *a* = additive effect, *d* = dominance effect, *ae*_1_ = additive by environment 1 (Urbana) specific effect, *ae*_2_ = additive by environment 2 (Aurora) specific effect, *ae*_3_ = additive by environment 3 (Clayton) specific effect, *ae*_4_ = additive by environment 4 (Homestead) specific effect. SE: standard error of predicted effect. *–Log*_*10*_*P*: minus *log*_*10*_ (experiment-wise *P*-value), *h*^2^: estimated heritability. Bold underlined SNPs are strongly associated with family structure and were not identified in the principal component adjusted model. SNPs written in regular word were detected in both base and principal component adjusted model. **Table S3.** Highly significant SNPs identified by using the PC adjusted model for four maize ear traits. Effect: *a* = additive effect, *d* = dominance effect, *ae*_1_ = additive by environment 1 (Urbana) specific effect, *ae*_2_ = additive by environment 2 (Aurora) specific effect, *ae*_3_ = additive by environment 3 (Clayton) specific effect, *ae*_4_ = additive by environment 4 (Homestead) specific effect. SE: standard error of predicted effect. *–Log*_*10*_*P*: minus *log*_*10*_ (experiment-wise *P*-value), *h*^2^: estimated heritability. Bold underlined SNPs were newly identified in the principal component adjusted models, which were not identified in the base model. SNPs written in regular word were detected in both base and principal component adjusted model. **Table S4.** Superior lines and hybrids predicted by using full genetic model for four ear traits of maize. GSL = general superior line for four locations, SL (+)1 = superior line for Urbana, SL (+)2 = superior line for Aurora, SL (+)3 = superior line for Clayton, SL (+)4 = superior line for Homestead; GSH = general superior hybrid line for four locations, SH (+)1 = superior hybrid for Urbana, SH (+)2 = superior hybrid for Aurora, SH (+)3 = superior hybrid for Clayton, SH (+)4 = superior hybrid four Homestead; (+) = positive genotypic value. **Fig. S1.** Tree map of gene ontology terms of the identified genes. Tree map was plotted using the generated R-script from Revigo. Different child terms were clustered into several parents’ gene ontology terms. **Fig. S2.** Expression data of the identified genes in Ear and Tassel retrieved from Maize inflorescence database. Tip of the ear (ear_tip), middle of the ear (ear_mid), base of the ear (ear_base), tassel at stage 1 (tassel_stg1), tassel at stage 2 (tassel_stg2), and tassel at stage 3 (tassel_stg3) from experiments in ear and tassel development series; wild type ear at 2 mm (wt_2mm_ear) from experiments in fascinated ear (fea4); and wild type ear 2–5 mm from experiments in knotted1. (A) Expression of a set of identified genes in Ear. We plotted heat map based on expression values of the genes with continuous color scale, where white color represents low expression, yellow represent mid-level expression and red color represents high expression And (B) Expression of a set of identified genes in Tassel. Here different color had same meaning as Fig. A.

## Data Availability

The genotype (ZeaGBSv2.3) data is accessible through CyVerse Discovery Environment (Data Store file path*: /iplant/home/shared/panzea/genotypes/GBS/v23/) and phenotypic data is available in https://www.panzea.org/phenotypes.

## Abbreviations

GWAS: Genome-wide association study
SNP: Single nucleotide polymorphism
NAM: Nested association mapping
GMDR: Generalized multifactor-factor dimensionality reduction
GPU: Graphical processing unit
GO: Gene ontology
SL: Superior line
SH: Superior hybrid line
GSL: General superior line
GE: Gene by environment interaction
