# Supplementary material for: Genome-wide association studies revealed complex genetic architecture and breeding perspective of maize ear traits
Source: BMC Plant Biol. 2022 Nov 18;22:537. doi: 10.1186/s12870-022-03913-1 (PMC9673299; doi:10.1186/s12870-022-03913-1)
Supplement: Supplementary file 1 — Additional file 1: Text S1. Details about the statistical models used for analyses. Table S1. Highly significant SNPs identified by using the base model with no G × E effects for four maize ear traits Effect: a = additive effect, d = dominance effect,. aa = additive × additive interaction effect, dd = dominance × dominance interaction effect. SE: standard error of estimated effect/predicted effect. –Log10P: minus log10 (P-value), h2: estimated heritability. Bold underlined SNPs are strongly associated with family structure and were not identified in the principal component adjusted model. SNPs written in regular word were detected in both base and principal component adjusted model. Table S2. Highly significant SNPs identified by using the base model with G × E effects for four maize ear traits. Effect: a = additive effect, d = dominance effect, ae1 = additive by environment 1 (Urbana) specific effect, ae2 = additive by environment 2 (Aurora) specific effect, ae3 = additive by environment 3 (Clayton) specific effect, ae4 = additive by environment 4 (Homestead) specific effect. SE: standard error of predicted effect. –Log10P: minus log10 (experiment-wise P-value), h2: estimated heritability. Bold underlined SNPs are strongly associated with family structure and were not identified in the principal component adjusted model. SNPs written in regular word were detected in both base and principal component adjusted model. Table S3. Highly significant SNPs identified by using the PC adjusted model for four maize ear traits. Effect: a = additive effect, d = dominance effect, ae1 = additive by environment 1 (Urbana) specific effect, ae2 = additive by environment 2 (Aurora) specific effect, ae3 = additive by environment 3 (Clayton) specific effect, ae4 = additive by environment 4 (Homestead) specific effect. SE: standard error of predicted effect. –Log10P: minus log10 (experiment-wise P-value), h2: estimated heritability. Bold underlined SNPs were newly identified in the prin [file 12870_2022_3913_MOESM1_ESM.docx]

**Text S1 Details about the statistical models used for analyses**

In the section of materials and methods, we briefly described the statistical approaches used in this study. The detailed process of *QTXNetwork* as follows,

**Individual locus detection** Significance testing was performed for each individual SNP locus in this step. To test the significance of the i-th individual SNP locus, we used the following linear mixed model,

$y_{hk}=\mu+a_{i}x_{A_{ik}}+d_{i}x_{D_{ik}}+e_{h}+ae_{ih}u_{AE_{ihk}}+de_{ih}u_{DE_{ihk}}+\varepsilon_{hk}$ (1)

where the definitions of parameters and coefficients are the same as what we defined in the materials and methods section of the main text.

Based on the model defined in equation 1, the F-test based on Henderson method III was used for significance testing. The locus with F-value exceeding the predefined threshold value is considered as a candidate individual SNP. To determine threshold value of the statistic, permutation testing was conducted by a total of 2,000 times for calculating the critical F-value to control the experiment-wise type I error (< 0.05).

**Epistasis loci detection** Based on the *m* significant candidates selected by step 1, a two-way interaction significance testing for all possible SNP pairs was performed. The model for testing the significance of epistasis interaction between the i-th SNP locus and j-th SNP locus can be written as

$\begin{matrix} y_{hk}=\mu+\sum_{i}^{m} (a_{i}x_{A_{ik}}+d_{i}x_{A_{ik}})+ aa_{ij}x_{AA_{ijk}}+ad_{ij}x_{AD_{ijk}}+da_{ij}x_{DA_{ijk}}+dd_{ij}x_{DD_{ijk}} \\ +e_{h}+\sum_{i}^{m} (ae_{ih}u_{AE_{ihk}}+de_{ih}u_{DE_{ihk}})+aae_{ijh}u_{AAE_{ijhk}}+ade_{ijh}u_{ADE_{ijhk}}+dae_{ijh}u_{DAE_{ijhk}}+dde_{ijh}u_{DD_{ijhk}}+\varepsilon_{hk} \end{matrix}$ (2)

where the definition of the all parameters and coefficients were the same as those in materials and methods sections of the main text. Based on the mixed epistasis models, the F-test was performed to test all possible SNP pairs. The pair of loci with F-value larger than the predefined threshold value is considered as candidate epistasis interacting loci. To determine threshold value, permutation testing was conducted by a total of 2,000 times for calculating the critical F-value to control the experiment-wise type I error ($\alpha_{EW}<$ 0.05).

**Model selection** In this step, the stepwise model selections were performed on all previously significantly detected individual and pair-wise epistasis loci. Therefore, we obtained an optimal set of candidate individual and pair-wise loci for the full genetic model,

$\begin{matrix} y_{hk}=\mu+\sum_{i} a_{i}x_{A_{ik}}+\sum_{i} d_{i}x_{D_{ik}}+\sum_{i<j} aa_{ij}x_{AA_{ijk}}+\sum_{i<j} ad_{ij}x_{AD_{ijk}}+\sum_{i<j} da_{ij}x_{DA_{ijk}}+\sum_{i<j} dd_{ij}x_{DD_{ijk}} \\ +e_{h}+\sum_{i} ae_{ih}u_{AE_{ihk}}+\sum_{i} de_{ih}u_{DE_{ihk}}+\sum_{i<j} aae_{ijh}u_{AAE_{ijhk}}+\sum_{i<j} ade_{ijh}u_{ADE_{ijhk}}+\sum_{i<j} dae_{ijh}u_{DAE_{ijhk}}+\sum_{i<j} dde_{ijh}u_{DD_{ijhk}}+\varepsilon_{hk} \end{matrix}$ (3)

A matrix form for the above mixed linear model equations can be expressed as,

(4)

where, $\mathbf{y}\sim MVN(\mathbf{Xb}, \sum\sigma_{v}^{2}\mathbf{U}_{v}\mathbf{U}_{v}^{T}+\mathbf{I}\sigma_{\varepsilon}^{2})$ is an n × 1 column vector of phenotypic values and *n* is the sample size of observations; $\mathbf{b}_{u}$is the *u*-th vector of fixed effects with known incidence matrix $\mathbf{X}_{u}$ ; $\mathbf{U}_{v}$ is the known coefficient matrix relating to the *v*-th random vector $\mathbf{e}_{v}$; $e_{v}\sim MVN(0,I\sigma_{v}^{2})$; and $\mathbf{e}_{\varepsilon}\sim MVN(\mathbf{0}, \mathbf{I}\sigma_{\varepsilon}^{2})$ is an n × 1 column vector of residual effects.

The linear mixed model and its distribution can be expressed in matrix notation,

$\begin{matrix} \mathbf{y}=\mathbf{1}\mu+\mathbf{X}_{A}\mathbf{b}_{A}+\mathbf{X}_{D}\mathbf{b}_{D}+\mathbf{X}_{AA}\mathbf{b}_{AA}+\mathbf{X}_{AD}\mathbf{b}_{AD}+\mathbf{X}_{DA}\mathbf{b}_{DA}+\mathbf{X}_{DD}\mathbf{b}_{DD} \\ +\mathbf{U}_{E}\mathbf{e}_{E}+\mathbf{U}_{AE}\mathbf{e}_{AE}+\mathbf{U}_{DE}\mathbf{e}_{DE}+\mathbf{U}_{AAE}\mathbf{e}_{AAE}+\mathbf{U}_{ADE}\mathbf{e}_{ADE}+\mathbf{U}_{DAE}\mathbf{e}_{DAE}+\mathbf{U}_{DDE}\mathbf{e}_{DDE}+\mathbf{e}_{\varepsilon} \\ =\mathbf{1}\mu+\sum_{u=1}^{6} \mathbf{X}_{u}\mathbf{b}_{u}+\sum_{v=1}^{7} \mathbf{U}_{v}\mathbf{e}_{v}+\mathbf{e}_{\varepsilon}\sim MVN\left(\mathbf{Xb},\sum_{v=1}^{6} \mathbf{U}_{v}\mathbf{V}_{v}\mathbf{U}_{v}^{T}+\mathbf{I}\sigma_{\varepsilon}^{2}\right) \end{matrix}$

where $\mathbf{y}$ is an *n × 1* column vector of phenotypic values and *n* is the sample size of observations; $\mu$ is the population mean, $\mathbf{b}_{u}$ is the $\mu$***-****th* vector of fixed effects; $\mathbf{X}_{u}$ is the known incidence matrix relating to the *u*-th fixed effects; $\mathbf{e}_{v}$is the *v*-th vector of random effects with distribution

$e_{v}\sim MVN(0, V_{v})$; $U_{v}$ is the known coefficient matrix for the *v*-th vector of random effects; and $e_{\varepsilon}\sim MVN(0, I\sigma_{\varepsilon}^{2})$ is a column vector of residual effects.

According to Luo *et al.* (2017) heritability of individual genetic effects is estimated by $h_{g}^{2}=\alpha\sigma_{g}^{2}/V_{P}$ ($\alpha=2$ for additive effect, $\alpha=1$ for dominant effect, $\alpha=4$for additive × additive, $\alpha=2$ for additive × dominant or dominant × additive, and $\alpha=1$ for dominant × dominant), where phenotypic variance ($V_{P}$) is the sum of genetic variance ($V_{G}$), genetic by environment interaction variance ($V_{GE}$), and residual variance ($V_{\varepsilon}$),

$$\begin{matrix} V_{P}=V_{G}+V_{GE}+V_{\varepsilon} \\ =\left(V_{A}+V_{D}+V_{I}\right)+\left(V_{AE}+V_{DE}+V_{IE}\right)+V_{\varepsilon} \\ =\left(V_{A}+V_{D}+V_{AA}+V_{AD}+V_{DA}+V_{DD}\right)+ \\ \left(V_{AE}+V_{DE}+V_{AAE}+V_{ADE}+V_{DAE}+V_{DDE}\right)+V_{\varepsilon} \end{matrix}$$

The total heritability is estimated by

$$\begin{matrix} h_{T}^{2}=h_{G}^{2}+h_{GE}^{2} \\ =(h_{A}^{2}+h_{D}^{2}+h_{AA}^{2}+h_{AD}^{2}+h_{DA}^{2}+h_{DD}^{2}) \\ +(h_{AE}^{2}+h_{DE}^{2}+h_{AAE}^{2}+h_{ADE}^{2}+h_{DAE}^{2}+h_{DDE}^{2}) \\ =(h_{A}^{2}+h_{D}^{2}+h_{I}^{2})+(h_{AE}^{2}+h_{DE}^{2}+h_{IE}^{2}) \end{matrix}$$

where $h_{T}^{2}$ is the total heritability; $h_{A}^{2}=\sum_{i} h_{a}^{2}$ is the heritability due to additive effects contributed by the sum of individual loci, $h_{D}^{2}=\sum_{i} h_{d}^{2}$ is the heritability due to dominance effects contributed by the sum of individual loci, $h_{AA}^{2}=\sum_{i<j} h_{aa}^{2}$is the heritability contributed by the sum of pair-wise additive by additive (*aa*) epistasis, $h_{AD}^{2}=\sum_{i<j} h_{ad}^{2}$ is the heritability contributed by the sum of pair-wise additive by dominance (*ad*) epistasis, $h_{DA}^{2}=\sum_{i<j} h_{da}^{2}$is the heritability contributed by the sum of pair-wise dominance by additive (*da*) epistasis, $h_{DD}^{2}=\sum_{i<j} h_{dd}^{2}$ is the heritability contributed by the sum of pair-wise dominance by dominance (*dd*) epistasis, $h_{AE}^{2}=\sum_{i} h_{ae}^{2}$ is additive by environment interaction heritability contributed by the sum of individual additive by environment interaction effects, $h_{DE}^{2}=\sum_{i} h_{de}^{2}$is dominance by environment interaction heritability contributed by the sum of individual dominance by environment interaction effects, $h_{AAE}^{2}=\sum_{i<j} h_{aae}^{2}$is *aa* epistasis by environment interaction heritability contributed by the sum of pair-wise *aa* epistasis by environment interaction effects, $h_{ADE}^{2}=\sum_{i<j} h_{ade}^{2}$is *ad* epistasis by environment interaction heritability contributed by the sum of pair-wise *ad* epistasis by environment interaction effects, $h_{DAE}^{2}=\sum_{i<j} h_{dae}^{2}$ is *da* epistasis by environment interaction heritability contributed by the sum of pair-wise *da* epistasis by environment interaction effects, $h_{DDE}^{2}=\sum_{i<j} h_{dde}^{2}$ is *dd* epistasis by environment interaction heritability contributed by the sum of pair-wise *dd* epistasis by environment interaction effects.

**Table S1** **Highly significant SNPs identified by using the base model with no G×E effects for four maize ear traits**

| **Trait** | **SNP** | **Gene** | **Effect** | **Estimate** | **SE** | ***–Log_10_P*** | ***h*^2^(%)** | **Gene Descriptions** |
| --- | --- | --- | --- | --- | --- | --- | --- | --- |
| **Weight** | **S1_8538065** | ***Zm00001d027588*** | *a* | –0.96 | 0.20 | 5.67 | 0.20 | Unknown |
|  | **S1_16698832** | ***Zm00001d027884*** | *a* | –0.99 | 0.20 | 5.90 | 0.21 | Copper-transporting ATPase PAA2 chloroplastic |
|  | **S1_31249633** | ***Zm00001d028339*** | *a* | 1.33 | 0.20 | 10.34 | 0.38 | Whole genome shotgun sequence of line PN40024 scaffold_7.assembly12x (Fragment) |
|  | **S1_228415652** | ***Zm00001d032497*** | *a* | 1.05 | 0.20 | 6.79 | 0.24 | Unknown |
|  | S2_14940618 | *Zm00001d002536* | *a* | –1.09 | 0.21 | 6.97 | 0.26 | Probable L-type lectin-domain containing receptor kinase S.5 |
|  | **S2_20018052** | ***Zm00001d002713*** | *a* | 1.25 | 0.20 | 9.10 | 0.34 | Vascular-related nac-domain 6 |
|  | S3_18999799 | *Zm00001d039908* | *a* | –1.90 | 0.20 | 20.32 | 0.79 | Ribosomal protein L26 |
|  | **S3_35554763** | ***Zm00001d040279*** | *a* | 1.45 | 0.20 | 12.25 | 0.46 | topless-related1 |
|  |  |  | *d* | –4.62 | 0.96 | 5.83 | 2.34 |  |
|  | **S3_196209906** | ***Zm00001d043317*** | *a* | –1.13 | 0.20 | 7.47 | 0.28 | Mitochondrial glycoprotein family protein |
|  | S3_208063573 | *Zm00001d043713* | *a* | 1.29 | 0.20 | 9.81 | 0.37 | plant/F18B13-26 protein |
|  | **S4_204960226** | ***Zm00001d052922*** | *a* | 1.42 | 0.20 | 11.54 | 0.44 | Unknown |
|  | S5_10493718 | *Zm00001d013405* | *a* | 1.96 | 0.20 | 21.75 | 0.84 | Protein kinase superfamily protein |
|  | **S5_30296160** | ***Zm00001d014039*** | *a* | –1.75 | 0.20 | 17.04 | 0.67 | non-intrinsic ABC protein 4 |
|  | **S5_33610966** | ***Zm00001d014132*** | *a* | 0.94 | 0.20 | 5.44 | 0.19 | Transcription factor-like protein DPB |
|  | S5_206846565 | *Zm00001d017788* | *a* | 1.44 | 0.20 | 11.88 | 0.45 | Dof zinc finger protein DOF2.1 |
|  | S6_137891608 | *Zm00001d037792* | *a* | 1.35 | 0.20 | 10.55 | 0.40 | Unknown |
|  | S10_4071219 | *Zm00001d023365* | *a* | 1.15 | 0.20 | 7.82 | 0.29 | Putative CBL-interacting protein kinase family protein |
|  | **S10_85581567** | ***Zm00001d024728*** | *a* | 1.21 | 0.20 | 8.70 | 0.32 | Unknown |
| **Length** | **S1_25374552** | ***Zm00001d028173*** | *a* | –3.07 | 0.18 | 67.76 | 1.79 | FRIGIDA-like protein 1 |
|  | **S1_31249633** | ***Zm00001d028339*** | *d* | –3.73 | 0.82 | 5.27 | 1.32 | Whole genome shotgun sequence of line PN40024 scaffold_7.assembly12x (Fragment) |
|  | **S1_230957070** | ***Zm00001d032572*** | *a* | 1.14 | 0.17 | 10.31 | 0.24 | LanC-like protein 2 |
|  | **S1_253269479** | ***Zm00001d033186*** | *d* | 2.97 | 0.65 | 5.31 | 0.84 | Mitochondrial import receptor subunit TOM5 homolog |
|  | **S1_262183867** | ***Zm00001d033407*** | *a* | 1.52 | 0.17 | 17.72 | 0.44 | Transcription factor bHLH28 |
|  | S1_286439897 | *Zm00001d034199* | *a* | –1.29 | 0.17 | 13.15 | 0.32 | Ocs element-binding factor 1 |
|  | **S2_25930299** | ***Zm00001d002889*** | *a* | 1.25 | 0.18 | 11.92 | 0.30 | Soluble inorganic pyrophosphatase |
|  | **S2_44200703** | ***Zm00001d003431*** | *a* | –1.13 | 0.18 | 9.91 | 0.24 | Pectinesterase 5 |
|  | **S4_133820555** | ***Zm00001d050963*** | *a* | –1.45 | 0.17 | 16.10 | 0.40 | Unknown |
|  | **S4_155296773** | ***Zm00001d051342*** | *a* | 0.78 | 0.17 | 5.12 | 0.12 | Chaperone DnaJ-domain superfamily protein |
|  | S5_206846565 | *Zm00001d017788* | *a* | 1.90 | 0.17 | 27.09 | 0.69 | Dof zinc finger protein DOF2.1 |
|  | S6_41511513 | *Zm00001d035693* | *a* | 1.05 | 0.17 | 8.85 | 0.21 | REX1 DNA Repair family protein |
|  | **S6_120848430** | ***Zm00001d037318*** | *a* | 1.38 | 0.18 | 14.35 | 0.36 | Putative AMP-dependent synthetase and ligase superfamily protein |
|  | **S6_129436296** | ***Zm00001d037557*** | *a* | 1.43 | 0.18 | 15.75 | 0.39 | E3 ubiquitin-protein ligase UPL6 |
|  | S8_71061430 | *Zm00001d009570* | *a* | 1.13 | 0.17 | 10.17 | 0.24 | Splicing factor U2af subunit isoform |
|  | S9_125805442 | *Zm00001d047293* | *a* | –1.37 | 0.18 | 14.14 | 0.36 | Glucan endo-1%2C3-beta-glucosidase 4 |
|  | S10_108059601 | *Zm00001d025182* | *a* | 2.62 | 0.17 | 50.36 | 1.30 | Protein XRI1 |
|  | **S1_25374552×**  **S1_31249633** | ***Zm00001d028173×***  ***Zm00001d028339*** | *aa* | 1.64 | 0.18 | 19.59 | 1.02 | FRIGIDA-like protein 1.  Whole genome shotgun sequence of line PN40024 scaffold_7.assembly12x (Fragment) |
|  |  |  | *da* | 6.25 | 0.78 | 15.04 | 7.42 |  |
|  | **S2_10886950×**  **S4_24978845** | ***Zm00001d004396×***  ***Zm00001d049295*** | *aa* | 1.30 | 0.18 | 12.33 | 0.64 | Vacuolar protein sorting-associated protein 54 chloroplastic |
|  |  |  | *da* | –5.96 | 0.61 | 21.76 | 6.76 | Auxin response factor 2 |
|  |  |  | *dd* | –7.21 | 1.53 | 5.61 | 4.94 |  |
| **Rank number** | **S1_15143038** | ***Zm00001d027850*** | *a* | 0.24 | 0.05 | 6.52 | 0.18 | P-loop containing nucleoside triphosphate hydrolases superfamily protein |
|  | **S1_26496034** | ***Zm00001d028211*** | *a* | –0.34 | 0.05 | 12.48 | 0.36 | Digalactosyldiacylglycerol synthase 2 chloroplastic |
|  | **S1_246485260** | ***Zm00001d033002*** | *a* | 0.22 | 0.05 | 5.65 | 0.15 | ATP-dependent 6-phosphofructokinase 5 chloroplastic |
|  | **S1_253656437** | ***Zm00001d033200*** | *a* | 0.24 | 0.05 | 6.57 | 0.19 | Gibberellin-regulated protein 1 |
|  | **S1_274718673** | ***Zm00001d033818*** | *a* | –0.30 | 0.05 | 10.18 | 0.28 | Transmembrane and coiled-coil domains protein 1 |
|  | S2_12602445 | *Zm00001d002435* | *a* | –0.33 | 0.05 | 12 | 0.36 | Alternative oxidase |
|  | **S2_132572194** | ***Zm00001d004708*** | *a* | –0.35 | 0.05 | 13.80 | 0.39 | F-box protein interaction domain containing protein |
|  | S2_208617113 | *Zm00001d006450* | *a* | 0.36 | 0.05 | 14.36 | 0.42 | UDP-glycosyltransferase 71B1 |
|  | S3_8300745 | *Zm00001d039580* | *a* | 0.33 | 0.05 | 12.46 | 0.35 | Ribosomal protein L18ae family |
|  | **S3_145178596** | ***Zm00001d041953*** | *a* | 0.20 | 0.05 | 5.04 | 0.13 | Unknown |
|  | **S3_149234811** | ***Zm00001d042060*** | *a* | –0.26 | 0.05 | 7.75 | 0.21 | AT-hook motif nuclear-localized protein 17 |
|  | S3_208048045 | *Zm00001d041953* | *a* | 0.29 | 0.05 | 9.84 | 0.28 | Unknown |
|  | **S4_58469273** | ***Zm00001d049993*** | *a* | –0.20 | 0.05 | 5.01 | 0.13 | Zinc transporter 3 |
|  | **S4_131765746** | ***Zm00001d050923*** | *a* | –0.21 | 0.05 | 5.47 | 0.15 | Ribosomal RNA processing Brix domain protein |
|  | S4_230754684 | *Zm00001d053415* | *a* | 0.31 | 0.05 | 10.83 | 0.31 | Unknown |
|  | **S5_10438838** | ***Zm00001d013400*** | *a* | 0.30 | 0.05 | 10.62 | 0.30 | Probable protein kinase |
|  | **S5_59431884** | ***Zm00001d014692*** | *a* | –0.37 | 0.05 | 15.26 | 0.44 | AIG2-like protein |
|  | S5_164655502 | *Zm00001d016493* | *a* | –0.29 | 0.05 | 9.27 | 0.27 | RS21-C6 protein |
|  | **S5_211731257** | ***Zm00001d018002*** | *a* | 0.33 | 0.05 | 12.28 | 0.36 | Unknown |
|  | **S6_89412349** | ***Zm00001d036468*** | *a* | 0.20 | 0.05 | 5.11 | 0.13 | Unknown |
|  | S6_138185969 | *Zm00001d037802* | *a* | –0.23 | 0.05 | 5.96 | 0.16 | Unknown |
|  | S7_157455909 | *Zm00001d021591* | *a* | 0.30 | 0.05 | 10.72 | 0.30 | Dof zinc finger protein DOF1.6 |
|  | S8_6850304 | *Zm00001d008380* | *a* | 0.34 | 0.05 | 12.30 | 0.37 | rRNA processing protein-related |
|  | **S8_113290875** | ***Zm00001d010408*** | *d* | 1.21 | 0.25 | 5.73 | 2.36 | Unknown |
|  | **S2_12602445×**  **S2_101662934** | ***Zm00001d002435×***  ***Zm00001d004300*** | *aa* | –0.23 | 0.04 | 6.16 | 0.36 | Alternative oxidase |
|  |  |  |  |  |  |  |  | E3 ubiquitin-protein ligase MBR2 |
|  | **S2_13257219×**  **S3_145178596** | ***Zm00001d004708×***  ***Zm00001d041953*** | *dd* | –3.54 | 0.40 | 18.03 | 20.23 | F-box protein interaction domain containing protein |
|  |  |  |  |  |  |  |  | Unknown |
| **Row number** | S1_25374551 | *Zm00001d028173* | *a* | 0.16 | 0.02 | 18.16 | 0.41 | FRIGIDA-like protein 1 |
|  | **S1_32157278** | ***Zm00001d028363*** | *a* | –0.14 | 0.02 | 14.83 | 0.32 | 65-kDamicrotubule-associated protein 6 |
|  | **S1_259301727** | ***Zm00001d033333*** | *a* | –0.09 | 0.02 | 6.75 | 0.14 | ARM repeat superfamily protein |
|  | S2_8291660 | *Zm00001d002211* | *a* | –0.14 | 0.02 | 15.52 | 0.34 | F-box only protein 7 |
|  | **S2_16940309** | ***Zm00001d002618*** | *a* | 0.09 | 0.02 | 6.60 | 0.14 | Dehydration-responsive element-binding protein 1D |
|  | **S2_25773012** | ***Zm00001d002885*** | *a* | –0.09 | 0.02 | 7.25 | 0.15 | Putative CCR4-associated factor 1 homolog 3 |
|  | S2_45755779 | *Zm00001d003488* | *a* | –0.12 | 0.02 | 11.22 | 0.25 | UDP-glycosyltransferase 85A7 |
|  | **S2_56927520** | ***Zm00001d003741*** | *a* | 0.10 | 0.02 | 7.49 | 0.16 | Unknown |
|  | **S2_64298570** | ***Zm00001d003871*** | *a* | 0.18 | 0.02 | 26.24 | 0.59 | AP2 domain containing protein |
|  | **S2_111205444** | ***Zm00001d004428*** | *a* | –0.11 | 0.02 | 8.91 | 0.19 | Unknown |
|  | **S2_189275601** | ***Zm00001d005804*** | *a* | –0.15 | 0.02 | 16.61 | 0.37 | Auxin-repressed protein |
|  | S2_222962641 | *Zm00001d007130* | *a* | –0.16 | 0.02 | 19.32 | 0.42 | Heavy metal transport/detoxification superfamily protein |
|  |  |  | *d* | –0.56 | 0.10 | 8.23 | 2.59 |  |
|  | S3_219197847 | *Zm00001d044102* | *a* | 0.20 | 0.02 | 30.43 | 0.69 | Zn-dependent exopeptidases superfamily protein |
|  | S4_11646594 | *Zm00001d048988* | *a* | –0.18 | 0.02 | 24.04 | 0.53 | Non-lysosomal glucosylceramidase |
|  | **S4_36026805** | ***Zm00001d049581*** | *a* | 0.13 | 0.02 | 11.96 | 0.26 | myb-like transcription factor family protein |
|  | **S4_152790903** | ***Zm00001d051307*** | *a* | –0.12 | 0.02 | 10.65 | 0.23 | Autophagy-related protein 13a |
|  | S5_3145164 | *Zm00001d013006* | *a* | 0.12 | 0.02 | 11.58 | 0.25 | DNA gyrase subunit A chloroplastic/mitochondrial |
|  | **S5_21351199** | ***Zm00001d013818*** | *a* | 0.26 | 0.02 | 48.88 | 1.13 | Tryptophan N-monooxygenase 2 |
|  | **S5_32090529** | ***Zm00001d014093*** | *a* | 0.09 | 0.02 | 7.03 | 0.15 | 3-ketoacyl-CoA thiolase 2 peroxisomal |
|  | S5_83861266 | *Zm00001d015306* | *a* | 0.23 | 0.02 | 36.84 | 0.84 | Probable arabinose 5-phosphate isomerase |
|  |  |  | *d* | 0.37 | 0.08 | 5.41 | 1.13 |  |
|  | **S6_121373751** | ***Zm00001d037327*** | *a* | 0.08 | 0.02 | 6.42 | 0.13 | Zinc finger protein CONSTANS-LIKE 7 |
|  | S8_168279628 | *Zm00001d012101* | *a* | –0.21 | 0.02 | 30.85 | 0.69 | E3 ubiquitin-protein ligase RGLG1 |
|  | S9_55272046 | *Zm00001d046020* | *a* | –0.12 | 0.02 | 10.07 | 0.22 | Putative VHS/GAT domain containing family protein |
|  | S10_139076223 | *Zm00001d026126* | *a* | –0.20 | 0.02 | 29.16 | 0.67 | Probable serine/threonine-protein kinase Cx32 chloroplastic |
|  | **S1_25374551×**  **S5_3145164** | ***Zm00001d028173×***  ***Zm00001d013006*** | *da* | 0.61 | 0.07 | 15.87 | 6.18 | FRIGIDA-like protein 1 |
|  |  |  |  |  |  |  |  | DNA gyrase subunit A chloroplastic/mitochondrial |
|  | **S2_22296264×**  **S5_3145164** | ***Zm00001d007130×***  ***Zm00001d013006*** | *dd* | 1.57 | 0.19 | 15.30 | 20.18 | Heavy metal transport/detoxification superfamily protein.  DNA gyrase subunit A chloroplastic/mitochondrial |
|  | **S3_21919784×**  **S5_83861266** | ***Zm00001d044102×***  ***Zm00001d015306*** | *aa* | –0.12 | 0.02 | 10.29 | 0.46 | Heavy metal transport/detoxification superfamily protein.  Probable arabinose 5-phosphate isomerase |

Effect: *a*= additive effect, *d*= dominance effect, *aa*= additive × additive interaction effect, *dd*= dominance × dominance interaction effect. SE: standard error of estimated effect/predicted effect. -*Log_10_P*: minus *log_10_* (*P*-value), *h^2^*: estimated heritability. Bold underlined SNPs are strongly associated with family structure and were not identified in the principal component adjusted model. SNPs written in regular word were detected in both base and principal component adjusted model.

**Table S2** **Highly significant SNPs identified by using the base model with G×E effects for four maize ear traits**

| **Traits** | **SNP** | **Gene** | **Effect** | **Estimate** | **SE** | **–*Log_10_P*** | ***h*^2^(%)** | **Gene Descriptions** |
| --- | --- | --- | --- | --- | --- | --- | --- | --- |
| **Weight** | **S1_161708627** | ***Zm00001d030813*** | *ae_1_* | –2.99 | 0.40 | 13.31 | 0.90 | Cycloartenol synthase |
|  | **S5_58007416** | ***Zm00001d014664*** | *a* | 3.47 | 0.20 | 63.52 | 2.63 | Protein chloroplast  import apparatus 2 |
|  |  |  | *d* | 3.72 | 0.75 | 6.14 | 1.51 |  |
|  |  |  | *ae_2_* | –2.65 | 0.41 | 10.01 | 1.49 |  |
|  |  |  | *ae_3_* | 2.57 | 0.40 | 10.09 |  |  |
|  | S7_169254965 | *Zm00001d022066* | *a* | -0.95 | 0.20 | 5.54 | 0.20 | LRR receptor-like  serine/threonine-protein kinase RPK2 |
|  |  |  | *ae_2_* | –3.23 | 0.41 | 14.69 | 1.12 |  |
|  |  |  | *ae_3_* | 1.91 | 0.39 | 5.92 |  |  |
| **Length** | S1_19633901 | *Zm00001d027994* | *a* | 0.88 | 0.18 | 6.27 | 0.15 | Myosin heavy chain-related protein |
|  |  |  | *ae_4_* | –1.51 | 0.33 | 5.23 | 0.31 |  |
|  | **S1_259219744** | ***Zm00001d033330*** | *ae_1_* | 2.63 | 0.35 | 13.22 | 0.65 | Cyclin-D5-1 |
|  | **S2_8291660** | ***Zm00001d002211*** | *ae_1_* | –1.75 | 0.34 | 6.50 | 0.27 | F-box only protein 7 |
|  | **S2_58766499** | ***Zm00001d003776*** | *ae_3_* | 1.55 | 0.32 | 6.07 | 0.27 | Beta-fructofuranosidase%2C insolubleisoenzyme 2 |
|  | S2_80723409 | *Zm00001d004079* | *a* | 0.83 | 0.18 | 5.44 | 0.13 | Lectin-domain containing receptor kinase A4.1 |
|  |  |  | *ae_1_* | –1.65 | 0.36 | 5.44 | 0.30 |  |
|  | **S2_84678243** | ***Zm00001d004127*** | *ae_4_* | 1.60 | 0.34 | 5.58 | 0.34 | Unknown |
|  | S4_13418209 | *Zm00001d049034* | *a* | –0.90 | 0.17 | 6.88 | 0.16 | Unknown |
|  |  |  | *ae_1_* | 2.39 | 0.35 | 11.25 | 0.50 |  |
|  | **S4_24978845** | ***Zm00001d049295*** | *a* | 0.80 | 0.17 | 5.41 | 0.12 | Auxin response factor 2 |
|  |  |  | *ae_2_* | –2.47 | 0.36 | 11.24 | 1.11 |  |
|  |  |  | *ae_3_* | 2.37 | 0.32 | 12.94 |  |  |
|  | **S4_36026805** | ***Zm00001d049581*** | *ae_4_* | –2.00 | 0.33 | 8.74 | 0.35 | myb-like transcription factor familyprotein |
|  | S4_208812132 | *Zm00001d053008* | *a* | 1.38 | 0.17 | 14.75 | 0.36 | Glyoxal oxidase |
|  |  |  | *ae_1_* | 2.54 | 0.35 | 12.32 | 0.58 |  |
|  | S5_14608995 | *Zm00001d013575* | *a* | 0.81 | 0.18 | 5.32 | 0.12 | Cyclin-dependent kinase G-1 |
|  |  |  | *ae_1_* | 1.77 | 0.35 | 6.33 | 0.31 |  |
|  | **S5_172810002** | ***Zm00001d016693*** | *a* | –0.93 | 0.17 | 7.21 | 0.17 | Protein LITTLE ZIPPER 3 |
|  |  |  | *ae_1_* | 1.85 | 0.34 | 7.11 | 0.40 |  |
|  | **S6_159381559** | ***Zm00001d038533*** | *ae_1_* | 1.54 | 0.34 | 5.09 | 0.25 | centromeric histone H3 |
|  | S7_168746883 | *Zm00001d022046* | *a* | 1.71 | 0.17 | 22.77 | 0.56 | SNF2 domain-containing  protein / helicase  domain-containing protein / HNH endonuclease domain-containing protein |
|  |  |  | *ae_1_* | 2.67 | 0.35 | 13.85 | 0.63 |  |
|  |  |  | *ae_2_* | –1.81 | 0.36 | 6.39 |  |  |
|  |  |  | *ae_4_* | –1.58 | 0.34 | 5.61 |  |  |
|  | **S10_59877496** | ***Zm00001d024256*** | *ae_1_* | 2.37 | 0.36 | 10.61 | 0.52 | F-box protein PP2-A13 |
| **Rank number** | S1_179980347 | *Zm00001d031158* | *ae_2_* | –0.47 | 0.09 | 6.45 | 0.41 | pathogenesis related protein5 |
|  | **S2_101662934** | ***Zm00001d004300*** | *ae_2_* | 0.42 | 0.09 | 5.47 | 0.38 | E3 ubiquitin-protein ligase MBR2 |
|  | **S5_25548909** | ***Zm00001d013944*** | *ae_2_* | –0.43 | 0.09 | 5.42 | 0.35 | unknown |
| **Row Number** | S2_55445579 | *Zm00001d003706* | *a* | –0.08 | 0.02 | 5.20 | 0.11 | unknown |
|  |  |  | *ae_1_* | 0.20 | 0.04 | 7.45 | 0.54 |  |
|  |  |  | *ae_2_* | –0.17 | 0.04 | 5.38 |  |  |
|  | **S4_44134120** | ***Zm00001d049774*** | *ae_1_* | 0.30 | 0.03 | 17.64 | 0.50 | BTB/POZ and MATH domain-containing protein 1 |
|  | S6_150466027 | *Zm00001d038171* | *a* | –0.11 | 0.02 | 9.84 | 0.21 | Ubiquitin-associated/translation elongation factor EF1B protein |
|  |  |  | *ae_4_* | 0.19 | 0.03 | 7.53 | 0.41 |  |

Effect: *a*= additive effect, *d*= dominance effect, *ae*_1_= additive by environment 1 (Urbana) specific effect, *ae*_2_= additive by environment 2 (Aurora) specific effect, *ae*_3_= additive by environment 3 (Clayton) specific effect, *ae*_4_= additive by environment 4 (Homestead) specific effect. SE: standard error of predicted effect. ***–Log_10_P***: minus *log_10_* (experiment-wise *P*-value), *h*^2^: estimated heritability. Bold underlined SNPs are strongly associated with family structure and were not identified in the principal component adjusted model. SNPs written in regular word were detected in both base and principal component adjusted model.

**Table S3 Highly significant SNPs identified by using the PC adjusted model for four maize ear traits**

| **Traits** | **SNP** | **Gene** | **Effect** | **Estimate** | **SE** | **–*Log10*P** | ***h*^2^(%)** | **Gene Descriptions** |
| --- | --- | --- | --- | --- | --- | --- | --- | --- |
| **Weight** | **S1_60832157** | **Zm00001d029183** | *ae_2_* | 2.0489 | 0.4172 | 6.041 | 0.0062 | Unknown |
|  | **S1_259223303** | **Zm00001d033278** | *a* | 1.0218 | 0.2177 | 5.569 | 0.0026 | Unknown |
|  | S2_14940618 | Zm00001d002536 | *a* | -1.6283 | 0.2172 | 13.17 | 0.0067 | Probable L-type  lectin-domain  containing receptor  kinase S.5 |
|  | S3_18999799 | Zm00001d039908 | *a* | -1.2519 | 0.2136 | 8.327 | 0.004 | Ribosomal protein  L26 |
|  | S3_208063573 | Zm00001d043713 | *a* | 1.6102 | 0.2141 | 13.246 | 0.0066 | plant/F18B13-26  protein |
|  | **S4_31578798** | **Zm00001d049462** | *a* | -1.5079 | 0.2167 | 11.452 | 0.0058 | Unknown |
|  |  |  | *ae_4_* | 1.8572 | 0.4167 | 5.078 | 0.005 |  |
|  | **S4_234419646** | **Zm00001d053425** | *a* | -2.2553 | 0.2153 | 24.872 | 0.0129 | Unknown |
|  | S5_10493718 | Zm00001d013405 | *a* | 2.3461 | 0.2128 | 27.465 | 0.014 | Protein kinase  superfamily protein |
|  | **S5_65110963** | **Zm00001d014841** | *a* | -1.2625 | 0.2156 | 8.318 | 0.004 | Unknown |
|  |  |  | *ae_1_* | -2.0275 | 0.4186 | 5.892 | 0.0095 |  |
|  |  |  | *ae_4_* | 1.843 | 0.4163 | 5.019 |  |  |
|  | S5_206846565 | Zm00001d017788 | *a* | 2.0318 | 0.2143 | 20.564 | 0.0105 | Dof zinc finger  protein DOF2.1 |
|  | S6_137891608 | Zm00001d037792 | *a* | 1.6293 | 0.2138 | 13.576 | 0.0067 | Unknown |
|  | S7_169254965 | Zm00001d022066 | *a* | -2.1539 | 0.2143 | 22.985 | 0.0118 | LRR receptor-like  serine/threonine-protein  kinase RPK2 |
|  |  |  | *ae_2_* | -3.6634 | 0.4282 | 16.901 | 0.0152 |  |
|  | **S8_73905364** | **Zm00001d009626** | *a* | 0.7895 | 0.2146 | 3.628 | 0.0016 | Unknown |
|  | S10_4071219 | Zm00001d023365 | *a* | 2.3091 | 0.2142 | 26.291 | 0.0135 | Putative CBL-interacting  protein kinase family protein |
|  |  |  | *ae_4_* | -2.0446 | 0.4118 | 6.16 | 0.0063 |  |
|  | **S2_14940618 ×**  **S9_150235386** | **Zm00001d002536×**  **Zm00001d047976** | *aa* | -1.6145 | 0.2213 | 12.514 | 0.0132 | Probable L-type  lectin-domain  containing receptor  kinase S.5 |
| **Length** | S1_19633901 | Zm00001d027994 | *a* | 2.4164 | 0.1892 | 36.476 | 0.006 | Myosin heavy  chain-related protein |
|  | **S1_286439897** | **Zm00001d034199** | *a* | -1.1318 | 0.1879 | 8.757 | 0.0013 | Ocs element-binding  factor 1 |
|  | **S2_36451587** | **Zm00001d003218** | *a* | 1.4597 | 0.1894 | 13.876 | 0.0022 | Unknown |
|  | S2_80723409 | Zm00001d004079 | *a* | 2.4119 | 0.1921 | 35.277 | 0.006 | Lectin-domain  containing receptor  kinase A4.1 |
|  | **S2_211212304** | **Zm00001d006570** | *a* | 1.2256 | 0.1887 | 10.072 | 0.0015 | Unknown |
|  | **S3_219197847** | **Zm00001d044102** | *ae_1_* | -1.8746 | 0.3717 | 6.335 | 0.0021 | Zn-dependent  exopeptidases  superfamily protein |
|  | S4_13418209 | Zm00001d049034 | *a* | -0.839 | 0.1879 | 5.096 | 7.00E-04 | Unknown |
|  |  |  | *ae_1_* | 4.0869 | 0.3845 | 25.593 | 0.0058 |  |
|  |  |  | *ae_4_* | -1.6604 | 0.3683 | 5.183 |  |  |
|  | S4_208812132 | Zm00001d053008 | *a* | 2.0222 | 0.1883 | 26.102 | 0.0042 | Glyoxal oxidase |
|  |  |  | *ae_1_* | 5.606 | 0.3869 | 46.611 | 0.0114 |  |
|  |  |  | *ae_3_* | -2.2421 | 0.3518 | 9.723 |  |  |
|  |  |  | *ae_4_* | -2.5081 | 0.3708 | 10.86 |  |  |
|  | S5_14608995 | Zm00001d013575 | *a* | 1.087 | 0.1907 | 7.916 | 0.0012 | Cyclin-dependent  kinase G-1 |
|  |  |  | *ae_1_* | 3.5372 | 0.3891 | 18.968 | 0.0064 |  |
|  |  |  | *ae_4_* | -2.0266 | 0.3733 | 7.243 |  |  |
|  | S5_206846565 | Zm00001d017788 | *a* | 1.9956 | 0.1887 | 25.353 | 0.0041 | Dof zinc finger  protein DOF2.1 |
|  | S6_41511513 | Zm00001d035693 | *a* | 1.9789 | 0.1879 | 25.137 | 0.004 | REX1 DNA  Repair family protein |
|  | S7_168746883 | Zm00001d022046 | *a* | 2.0972 | 0.1867 | 28.459 | 0.0045 | SNF2 domain-containing  protein / helicase  domain-containing  protein / HNH  endonuclease  domain-containing protein |
|  |  |  | *ae_1_* | 4.8085 | 0.3832 | 35.261 | 0.0105 |  |
|  |  |  | *ae_2_* | -3.2661 | 0.3911 | 16.145 |  |  |
|  |  |  | *ae_4_* | -2.4586 | 0.3679 | 10.619 |  |  |
|  | S8_71061430 | Zm00001d009570 | *a* | 1.5062 | 0.1876 | 14.991 | 0.0023 | Splicing factor U2af  subunit isoform |
|  |  |  | *ae_4_* | -1.6715 | 0.3571 | 5.541 | 0.0023 |  |
|  | S9_125805442 | Zm00001d047293 | *a* | -1.3985 | 0.1911 | 12.589 | 0.002 | Glucan endo-1%2C3-  beta-glucosidase 4 |
|  | S10_108059601 | Zm00001d025182 | *a* | 2.7001 | 0.1882 | 45.73 | 0.0075 | Protein XRI1 |
|  | **S2_80723409 ×**  **S8_71061430** | **Zm00001d004079**  **× Zm00001d009570** | *dd* | -23.859 | 4.5412 | 6.822 | 0.2921 | Lectin-domain  containing receptor  kinase A4.1;  Splicing factor U2af  subunit isoform |
| **Rank Number** | S1_179980347 | Zm00001d031158 | *a* | -0.3102 | 0.049 | 9.616 | 0.0049 | pathogenesis related protein5 |
|  |  |  | *ae_2_* | -0.6308 | 0.0994 | 9.645 | 0.0127 |  |
|  | S2_12602445 | Zm00001d002435 | *a* | -0.5528 | 0.0497 | 27.921 | 0.0156 | Alternative oxidase |
|  |  |  | *ae_4_* | 0.4438 | 0.0953 | 5.494 | 0.0075 |  |
|  | S2_208617113 | Zm00001d006450 | *a* | 0.4902 | 0.0493 | 22.499 | 0.0122 | UDP-glycosyltransferase 71B1 |
|  | S3_8300745 | Zm00001d039580 | *a* | 0.4213 | 0.0488 | 17.215 | 0.009 | Ribosomal protein L18ae family |
|  | S3_208048045 | Zm00001d041953 | *a* | 0.3298 | 0.0491 | 10.737 | 0.0055 | Unknown |
|  | S4_230754684 | Zm00001d053415 | *a* | 0.327 | 0.0493 | 10.462 | 0.0054 | Unknown |
|  |  |  | *ae_1_* | 0.4197 | 0.0934 | 5.149 | 0.0062 |  |
|  | S5_164655502 | Zm00001d016493 | *a* | -0.4322 | 0.05 | 17.24 | 0.0095 | RS21-C6 protein |
|  |  |  | *ae_1_* | 0.5041 | 0.097 | 6.687 | 0.0085 |  |
|  |  |  | *ae_4_* | -0.4603 | 0.0977 | 5.603 |  |  |
|  | S6_138185969 | Zm00001d037802 | *a* | -0.3486 | 0.0495 | 11.701 | 0.0062 | Unknown |
|  | S7_157455909 | Zm00001d021591 | *a* | 0.463 | 0.0488 | 20.6 | 0.0109 | Dof zinc finger protein DOF1.6 |
|  | S8_6850304 | Zm00001d008380 | *a* | 0.4818 | 0.0501 | 21.14 | 0.0118 | rRNA processing protein-related |
|  |  |  | *ae_1_* | -0.4407 | 0.0948 | 5.476 | 0.0066 |  |
|  |  |  | *ae_3_* | 0.4209 | 0.0932 | 5.203 |  |  |
|  | **S9_150868664** | **Zm00001d047994** | *a* | -0.3413 | 0.0498 | 11.115 | 0.0059 | Unknown |
|  |  |  | *ae_1_* | -0.47 | 0.0954 | 6.075 | 0.0085 |  |
|  |  |  | *ae_4_* | 0.5202 | 0.0952 | 7.329 |  |  |
|  | **S1_274718673**  **× S8_140192740** | **Zm00001d033818**  **× Zm00001d000226** | *aa* | -0.3253 | 0.0493 | 10.351 | 0.0108 | Unknown*Transmembrane  and coiled-coil domains protein 1 |
| **Row Number** | S1_25374551 | Zm00001d028173 | *a* | 0.1616 | 0.0194 | 16.057 | 0.0051 | Unknown |
|  | S2_8291660 | Zm00001d002211 | *a* | -0.1283 | 0.0192 | 10.641 | 0.0032 | Unknown |
|  | S2_45755779 | Zm00001d003488 | *a* | -0.3254 | 0.0194 | 61.992 | 0.0207 | UDP-glycosyltransferase 85A7 |
|  | S2_222962641 | Zm00001d007130 | *a* | -0.1453 | 0.0191 | 13.467 | 0.0041 | Heavy metal transport/  detoxification superfamily  protein |
|  | S3_219197847 | Zm00001d044102 | *a* | 0.1711 | 0.0192 | 18.356 | 0.0057 | Zn-dependent exopeptidases  superfamily protein |
|  | S4_11646594 | Zm00001d048988 | *a* | -0.1776 | 0.019 | 19.929 | 0.0062 | Non-lysosomal  glucosylceramidase |
|  | S5_3145164 | Zm00001d013006 | *a* | 0.1896 | 0.0191 | 22.378 | 0.007 | DNA gyrase subunit  A chloroplastic/mitochondrial |
|  | S5_83861266 | Zm00001d015306 | *a* | 0.2834 | 0.0192 | 48.204 | 0.0157 | Probable arabinose  5-phosphate isomerase |
|  | S6_150466027 | Zm00001d038171 | *a* | -0.1948 | 0.0191 | 23.742 | 0.0074 | Ubiquitin-associated/  translation elongation  factor EF1B protein |
|  |  |  | *ae_4_* | 0.2337 | 0.0368 | 9.678 | 0.005 |  |
|  | S8_168279628 | Zm00001d012101 | *a* | -0.268 | 0.0191 | 43.623 | 0.014 | E3 ubiquitin-protein  ligase RGLG1 |
|  | S9_55272046 | Zm00001d046020 | *a* | -0.173 | 0.0193 | 18.494 | 0.0059 | Putative VHS/GAT  domain containing  family protein |
|  | S10_139076223 | Zm00001d026126 | *a* | -0.1869 | 0.0193 | 21.316 | 0.0068 | Probable serine/threonine-  protein kinase Cx32 chloroplastic |
|  | **S1_25374551**  **× S5_83861266** | **Zm00001d028173**  **× Zm00001d015306** | *da* | 0.5741 | 0.0912 | 9.5 | 0.0645 | Unknown* DNA  gyrase subunit A  chloroplastic/mitochondrial |

Effect: *a*= additive effect, *d*= dominance effect, *ae*_1_= additive by environment 1 (Urbana) specific effect, *ae*_2_= additive by environment 2 (Aurora) specific effect, *ae*_3_= additive by environment 3 (Clayton) specific effect, *ae*_4_= additive by environment 4 (Homestead) specific effect. SE: standard error of predicted effect. ***–Log_10_P***: minus *log_10_* (experiment-wise *P*-value), *h*^2^: estimated heritability. Bold underlined SNPs were newly identified in the principal component adjusted models, which were not identified in the base model. SNPs written in regular word were detected in both base and principal component adjusted model.

**Table S4** Superior lines and hybrids predicted by using full genetic model for four ear traits of maize

| **Weight** | **Z004E0030** | **GSL(+)** | **SL(+)1** | **SL(+)2** | **SL(+)3** | **SL(+)4** | **GSH(+)** | **SH(+)1** | **SH(+)2** | **SH(+)3** | **SH(+)4** |
| --- | --- | --- | --- | --- | --- | --- | --- | --- | --- | --- | --- |
| S2_14940618 | *Qq* | *qq* | *qq* | *qq* | *qq* | *qq* | *qq* | *qq* | *qq* | *qq* | *qq* |
| S3_18999799 | *QQ* | *qq* | *qq* | *qq* | *qq* | *qq* | *qq* | *qq* | *qq* | *qq* | *qq* |
| S4_204960226 | *QQ* | *QQ* | *QQ* | *QQ* | *QQ* | *QQ* | *Qq* | *Qq* | *QQ* | *Qq* | *Qq* |
| S5_10493718 | *Qq* | *QQ* | *QQ* | *QQ* | *QQ* | *QQ* | *QQ* | *QQ* | *QQ* | *QQ* | *QQ* |
| S5_30296160 | *Qq* | *QQ* | *qq* | *QQ* | *QQ* | *QQ* | *QQ* | *qq* | *QQ* | *QQ* | *QQ* |
| S5_58007416 | *QQ* | *QQ* | *QQ* | *QQ* | *QQ* | *QQ* | *Qq* | *Qq* | *Qq* | *QQ* | *Qq* |
| S5_206846565 | *Qq* | *QQ* | *QQ* | *QQ* | *QQ* | *QQ* | *QQ* | *QQ* | *QQ* | *QQ* | *QQ* |
| S7_169254965 | *Qq* | *QQ* | *QQ* | *qq* | *QQ* | *QQ* | *QQ* | *QQ* | *qq* | *QQ* | *QQ* |
| **Length** | **Z006E0233** | **GSL(+)** | **SL(+)1** | **SL(+)2** | **SL(+)3** | **SL(+)4** | **GSH(+)** | **SH(+)1** | **SH(+)2** | **SH(+)3** | **SH(+)4** |
| S1_31249633 | *QQ* | *qq* | *qq* | *qq* | *qq* | *qq* | *qq* | *qq* | *qq* | *qq* | *qq* |
| S1_230957070 | *qq* | *QQ* | *QQ* | *QQ* | *QQ* | *QQ* | *QQ* | *QQ* | *QQ* | *QQ* | *QQ* |
| S1_253269479 | *QQ* | *QQ* | *QQ* | *qq* | *QQ* | *QQ* | *Qq* | *Qq* | *Qq* | *Qq* | *Qq* |
| S2_25930299 | *Qq* | *QQ* | *QQ* | *QQ* | *QQ* | *QQ* | *QQ* | *QQ* | *QQ* | *QQ* | *QQ* |
| S2_44200703 | *Qq* | *qq* | *qq* | *qq* | *qq* | *qq* | *qq* | *qq* | *qq* | *qq* | *qq* |
| S2_58766499 | *qq* | *QQ* | *QQ* | *QQ* | *QQ* | *QQ* | *QQ* | *QQ* | *QQ* | *QQ* | *QQ* |
| S2_80723409 | *Qq* | *QQ* | *qq* | *QQ* | *QQ* | *QQ* | *QQ* | *qq* | *QQ* | *QQ* | *QQ* |
| S2_84678243 | *Qq* | *QQ* | *QQ* | *qq* | *QQ* | *QQ* | *QQ* | *QQ* | *qq* | *QQ* | *QQ* |
| S2_108869506 | *Qq* | *QQ* | *QQ* | *qq* | *QQ* | *QQ* | *QQ* | *QQ* | *Qq* | *QQ* | *QQ* |
| S4_13418209 | *qq* | *QQ* | *QQ* | *QQ* | *QQ* | *qq* | *QQ* | *QQ* | *QQ* | *QQ* | *qq* |
| S4_24978845 | *qq* | *QQ* | *QQ* | *qq* | *QQ* | *QQ* | *QQ* | *QQ* | *qq* | *QQ* | *QQ* |
| S4_133820555 | *QQ* | *qq* | *qq* | *qq* | *qq* | *qq* | *qq* | *qq* | *qq* | *qq* | *qq* |
| S4_155296773 | *qq* | *QQ* | *QQ* | *QQ* | *QQ* | *QQ* | *QQ* | *QQ* | *QQ* | *QQ* | *QQ* |
| S4_208812132 | *QQ* | *QQ* | *QQ* | *QQ* | *QQ* | *qq* | *Qq* | *QQ* | *Qq* | *Qq* | *Qq* |
| S5_14608995 | *QQ* | *QQ* | *QQ* | *QQ* | *QQ* | *QQ* | *Qq* | *QQ* | *Qq* | *Qq* | *Qq* |
| S6_129436296 | *QQ* | *QQ* | *QQ* | *QQ* | *QQ* | *QQ* | *Qq* | *QQ* | *Qq* | *Qq* | *Qq* |
| S6_159381559 | *qq* | *QQ* | *QQ* | *QQ* | *QQ* | *QQ* | *QQ* | *QQ* | *QQ* | *QQ* | *QQ* |
| S8_71061430 | *qq* | *QQ* | *QQ* | *QQ* | *QQ* | *qq* | *QQ* | *QQ* | *QQ* | *QQ* | *qq* |
| **Rank Number** | **Z016E0164** | **GSL(+)** | **SL(+)1** | **SL(+)2** | **SL(+)3** | **SL(+)4** | **GSH(+)** | **SH(+)1** | **SH(+)2** | **SH(+)3** | **SH(+)4** |
| S1_246485260 | *qq* | *QQ* | *QQ* | *QQ* | *QQ* | *QQ* | *QQ* | *QQ* | *QQ* | *QQ* | *QQ* |
| S2_132572194 | *Qq* | *qq* | *qq* | *qq* | *qq* | *qq* | *qq* | *qq* | *qq* | *qq* | *Qq* |
| S3_145178596 | *QQ* | *QQ* | *QQ* | *QQ* | *QQ* | *qq* | *Qq* | *Qq* | *Qq* | *Qq* | *qq* |
| S4_131765746 | *qq* | *QQ* | *qq* | *QQ* | *QQ* | *QQ* | *QQ* | *qq* | *QQ* | *QQ* | *QQ* |
| S5_164655502 | *qq* | *qq* | *qq* | *qq* | *qq* | *qq* | *Qq* | *Qq* | *Qq* | *Qq* | *Qq* |
| S6_89412349 | *QQ* | *QQ* | *QQ* | *QQ* | *QQ* | *QQ* | *Qq* | *Qq* | *Qq* | *Qq* | *Qq* |
| S6_138185969 | *qq* | *QQ* | *QQ* | *QQ* | *QQ* | *QQ* | *QQ* | *QQ* | *QQ* | *QQ* | *QQ* |
| S8_113290875 | *qq* | *QQ* | *QQ* | *QQ* | *QQ* | *QQ* | *Qq* | *Qq* | *Qq* | *Qq* | *Qq* |
| S4_58469273 | *qq* | *QQ* | *QQ* | *qq* | *QQ* | *QQ* | *QQ* | *QQ* | *qq* | *QQ* | *QQ* |
| **Row Number** | **Z019E0115** | **GSL(+)** | **SL(+)1** | **SL(+)2** | **SL(+)3** | **SL(+)4** | **GSH(+)** | **SH(+)1** | **SH(+)2** | **SH(+)3** | **SH(+)4** |
| S1_25374551 | *QQ* | *QQ* | *QQ* | *QQ* | *QQ* | *QQ* | *Qq* | *Qq* | *Qq* | *Qq* | *Qq* |
| S2_8291660 | *Qq* | *qq* | *qq* | *qq* | *qq* | *qq* | *qq* | *qq* | *qq* | *qq* | *qq* |
| S2_16940309 | *Qq* | *QQ* | *QQ* | *QQ* | *QQ* | *QQ* | *QQ* | *QQ* | *QQ* | *QQ* | *QQ* |
| S2_25773012 | *Qq* | *QQ* | *QQ* | *QQ* | *QQ* | *QQ* | *QQ* | *QQ* | *QQ* | *QQ* | *QQ* |
| S2_55445579 | *qq* | *QQ* | *QQ* | *qq* | *QQ* | *QQ* | *QQ* | *QQ* | *qq* | *QQ* | *QQ* |
| S2_56927520 | *qq* | *QQ* | *QQ* | *QQ* | *QQ* | *QQ* | *QQ* | *QQ* | *QQ* | *QQ* | *QQ* |
| S2_111205444 | *qq* | *QQ* | *QQ* | *QQ* | *QQ* | *QQ* | *QQ* | *QQ* | *QQ* | *QQ* | *QQ* |
| S2_189275601 | *Qq* | *qq* | *qq* | *qq* | *qq* | *qq* | *qq* | *qq* | *qq* | *qq* | *qq* |
| S2_222962641 | *Qq* | *qq* | *qq* | *qq* | *qq* | *qq* | *qq* | *Qq* | *qq* | *qq* | *qq* |
| S4_44134120 | *Qq* | *QQ* | *QQ* | *QQ* | *QQ* | *qq* | *QQ* | *QQ* | *QQ* | *QQ* | *qq* |
| S4_152790903 | *QQ* | *qq* | *qq* | *qq* | *qq* | *qq* | *qq* | *qq* | *qq* | *qq* | *qq* |
| S5_3145164 | *Qq* | *QQ* | *QQ* | *QQ* | *QQ* | *QQ* | *QQ* | *QQ* | *QQ* | *QQ* | *QQ* |
| S5_21351199 | *Qq* | *QQ* | *QQ* | *QQ* | *QQ* | *QQ* | *QQ* | *QQ* | *QQ* | *QQ* | *QQ* |
| S5_32090529 | *Qq* | *QQ* | *QQ* | *QQ* | *QQ* | *QQ* | *QQ* | *QQ* | *QQ* | *QQ* | *QQ* |
| S5_83861266 | *Qq* | *QQ* | *QQ* | *QQ* | *QQ* | *QQ* | *QQ* | *QQ* | *QQ* | *QQ* | *QQ* |
| S6_121373751 | *qq* | *QQ* | *QQ* | *QQ* | *QQ* | *QQ* | *QQ* | *QQ* | *QQ* | *QQ* | *QQ* |
| S6_150466027 | *Qq* | *qq* | *qq* | *qq* | *qq* | *QQ* | *qq* | *qq* | *qq* | *qq* | *QQ* |
| S8_168279628 | *QQ* | *qq* | *qq* | *qq* | *qq* | *qq* | *qq* | *qq* | *qq* | *qq* | *qq* |
| S9_55272046 | *QQ* | *qq* | *qq* | *qq* | *qq* | *qq* | *qq* | *qq* | *qq* | *qq* | *qq* |
| S10_139076223 | *Qq* | *qq* | *qq* | *qq* | *qq* | *qq* | *qq* | *qq* | *qq* | *qq* | *qq* |

GSL= general superior line for four locations, SL (+)1= superior line for Urbana, SL (+)2= superior line for Aurora, SL (+)3= superior line for Clayton, SL (+)4= superior line for Homestead; GSH= general superior hybrid line for four locations, SH (+)1= superior hybrid for Urbana, SH (+)2= superior hybrid for Aurora, SH (+)3= superior hybrid for Clayton, SH (+)4= superior hybrid four Homestead; (+)= positive genotypic value.


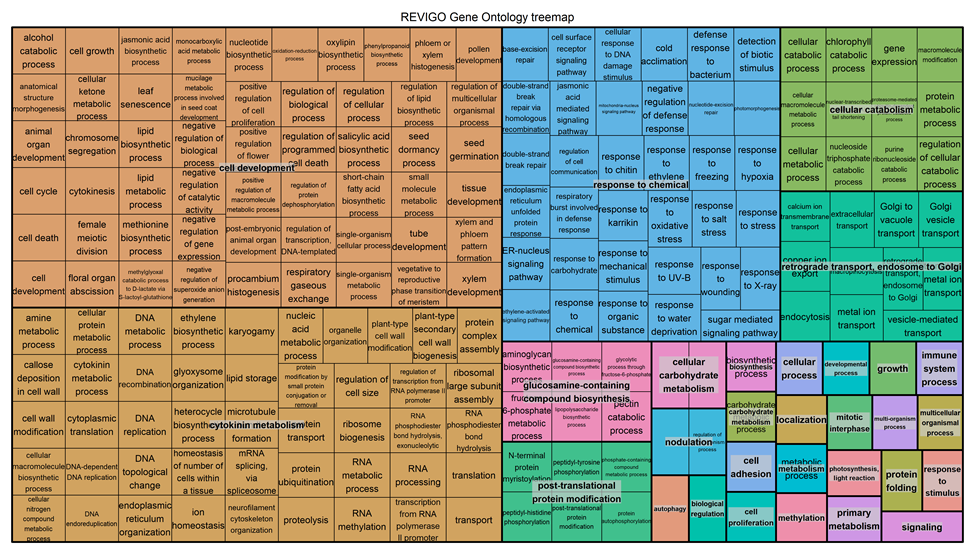


**Fig. S1 Tree map of gene ontology terms of the identified genes.** Tree map was plotted using the generated R-script from Revigo. Different child terms were clustered in several parents’ gene ontology terms for Biological process.


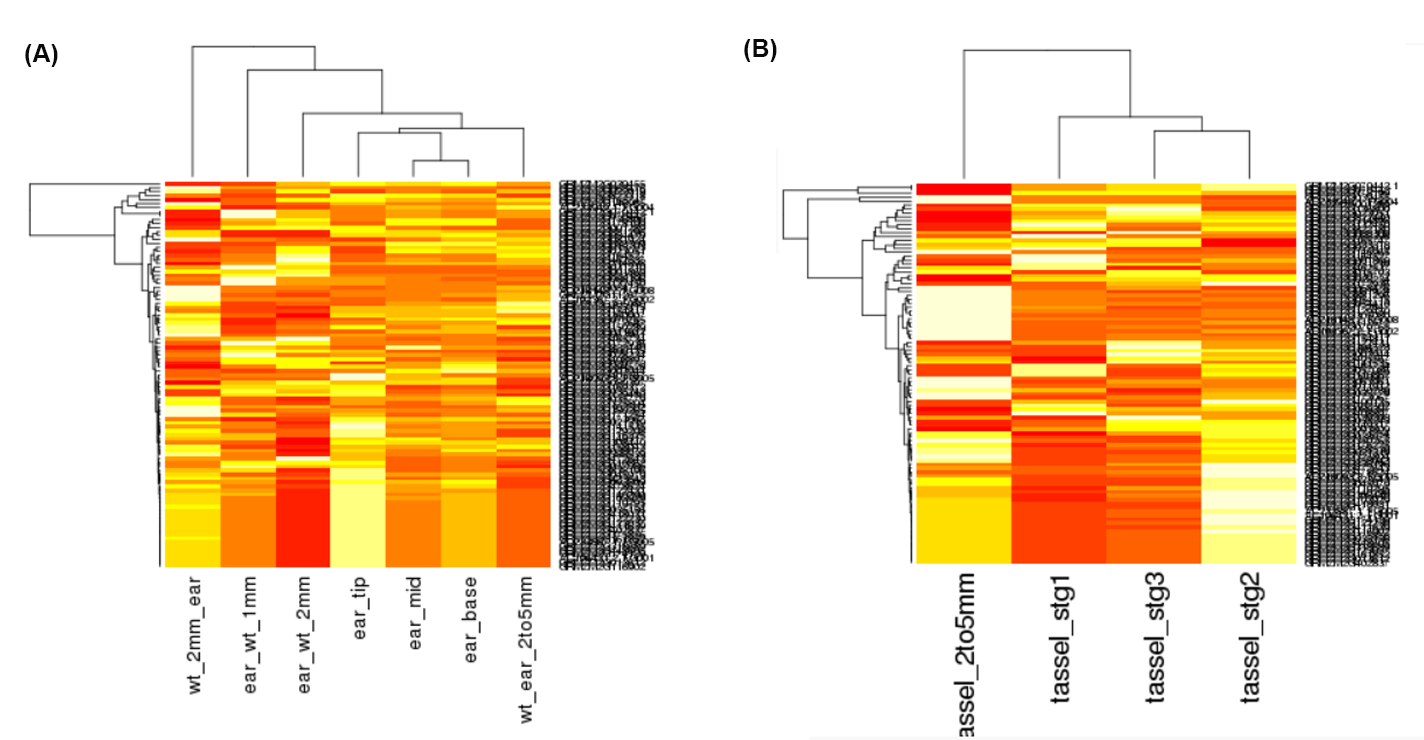


**Fig S2** **Expression data of the identified genes in Ear and Tassel retrieved from Maize inflorescence database**. (A) Expression of a set of identified genes in Ear. White color represents low expression, yellow represent mid-level expression and red color represents high expression. And (B) Expression of a set of identified genes in Tassel. Here different color had same meaning as fig A.
